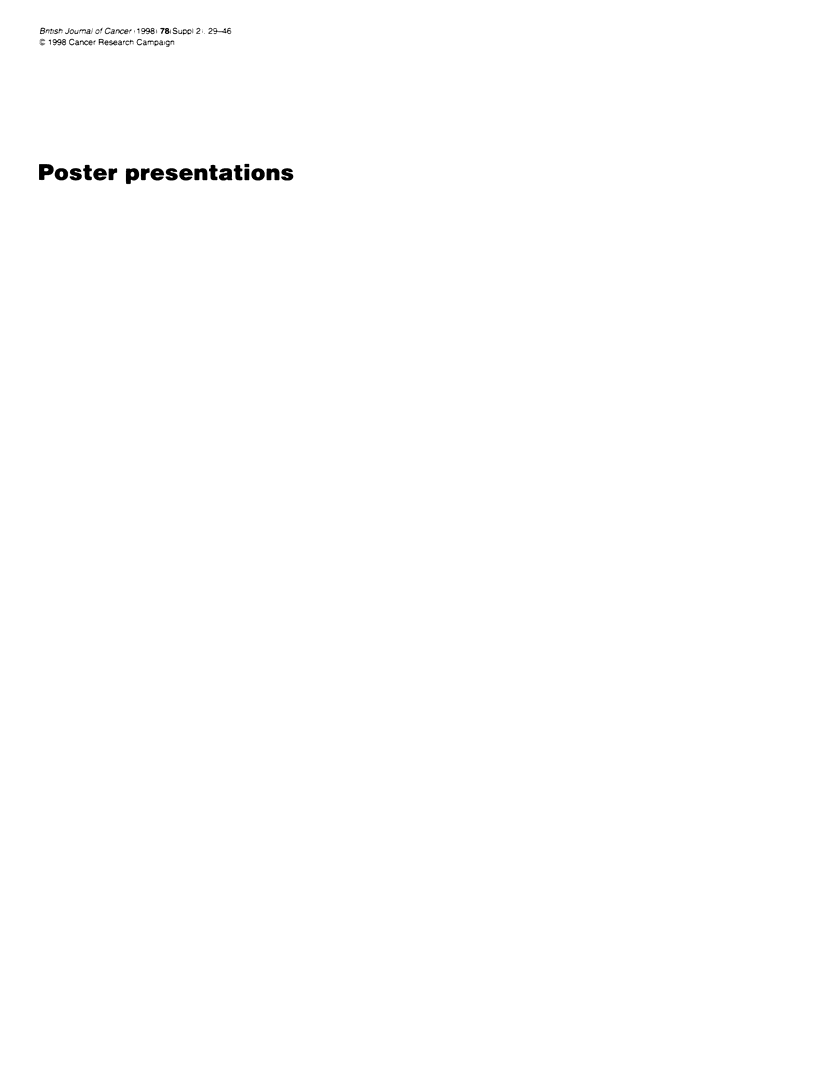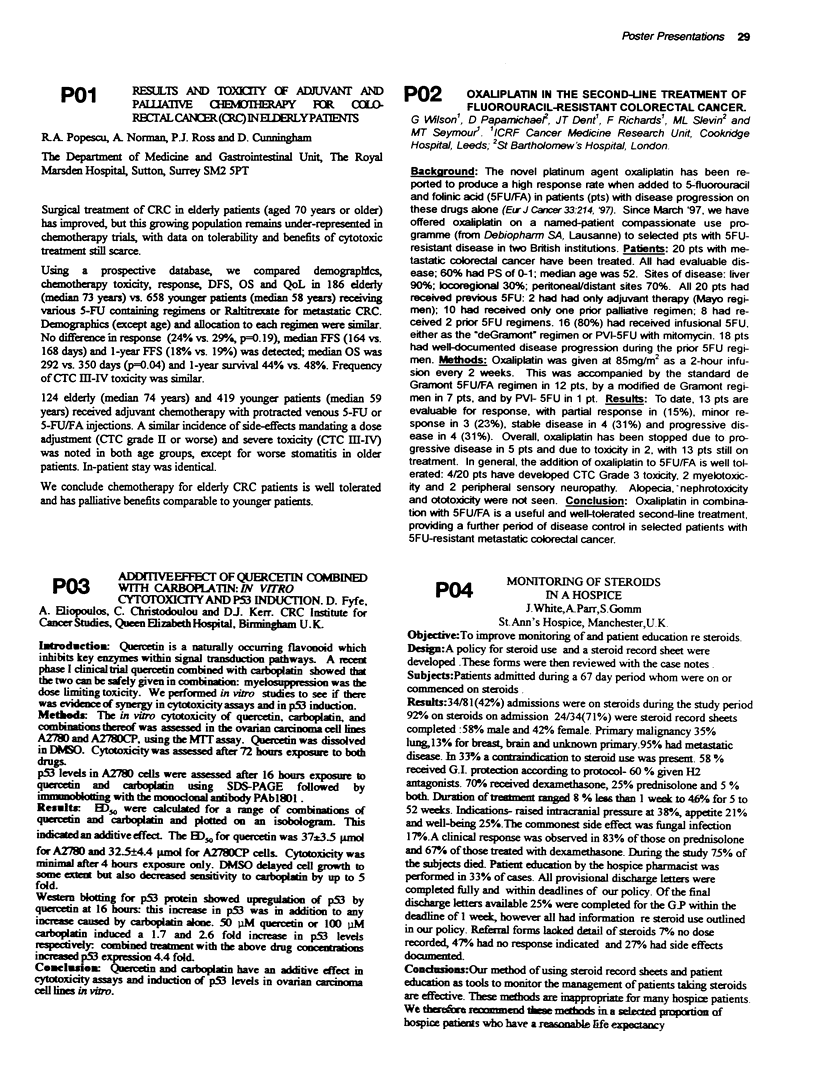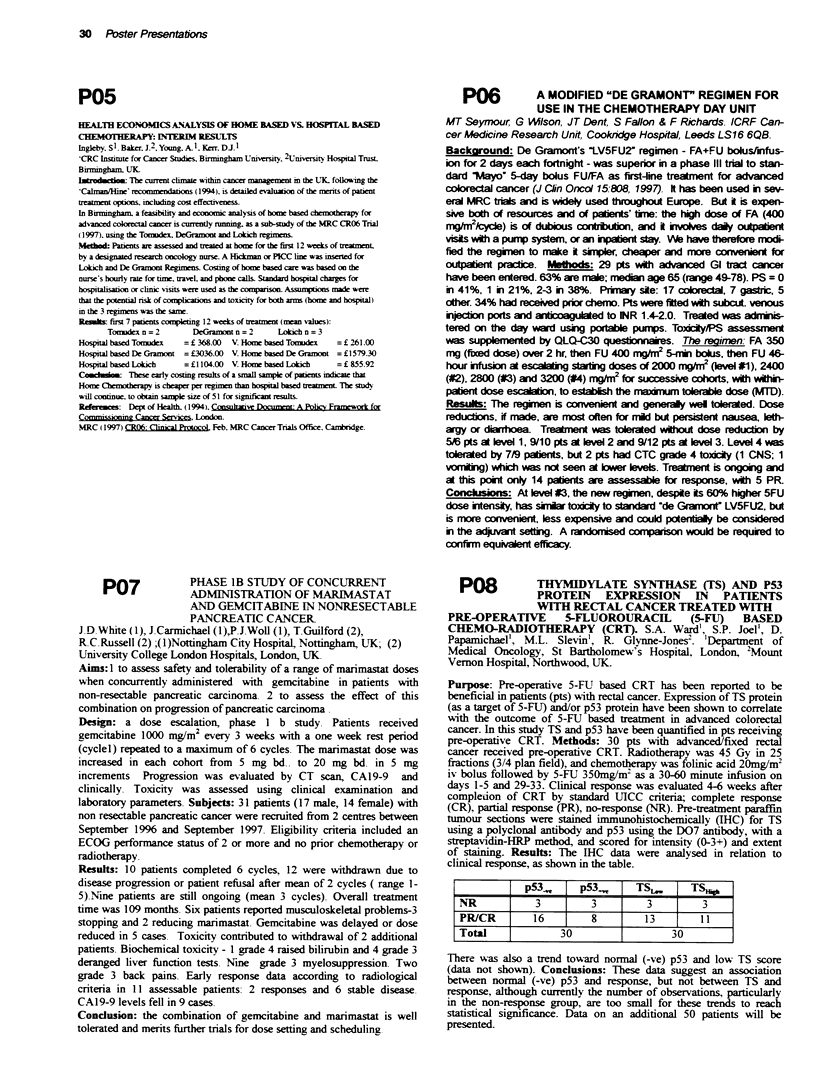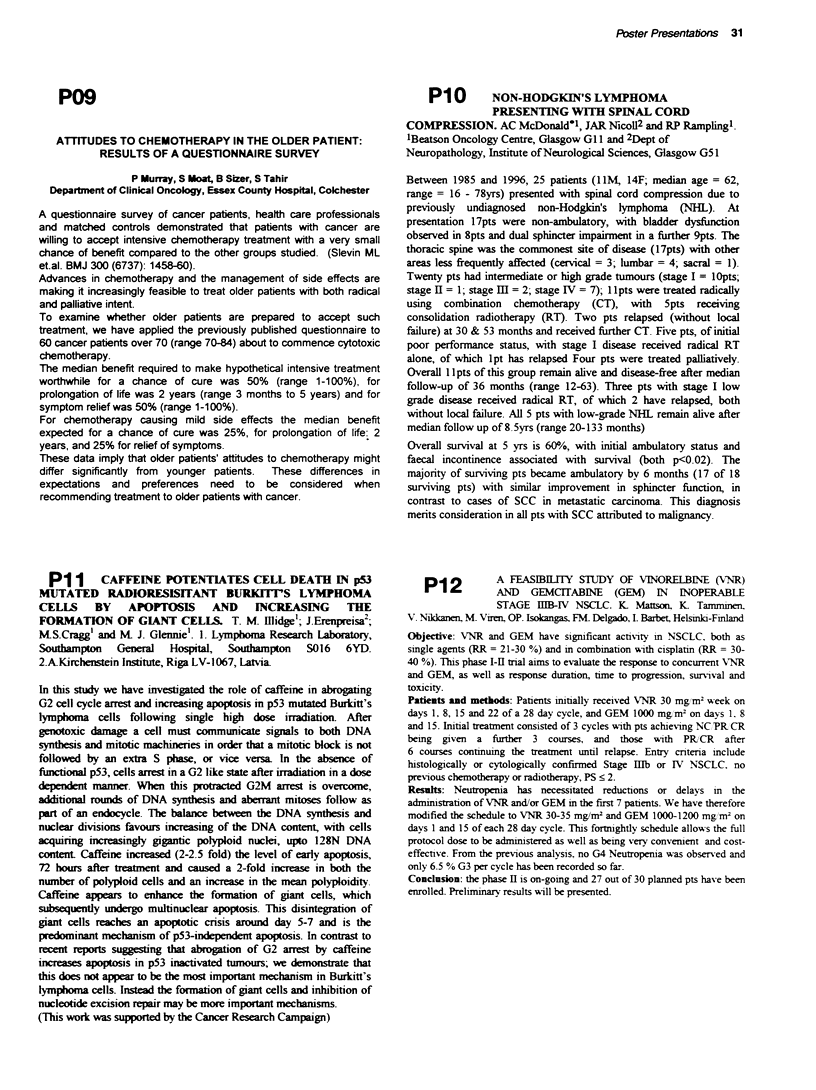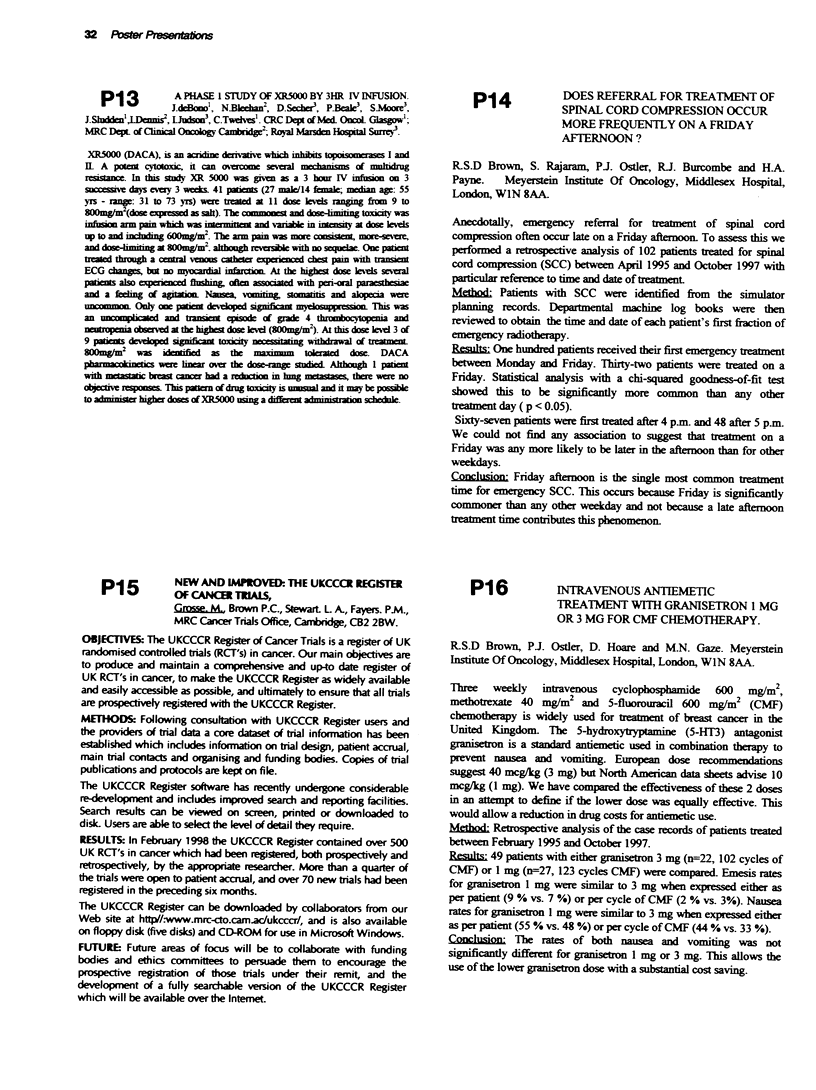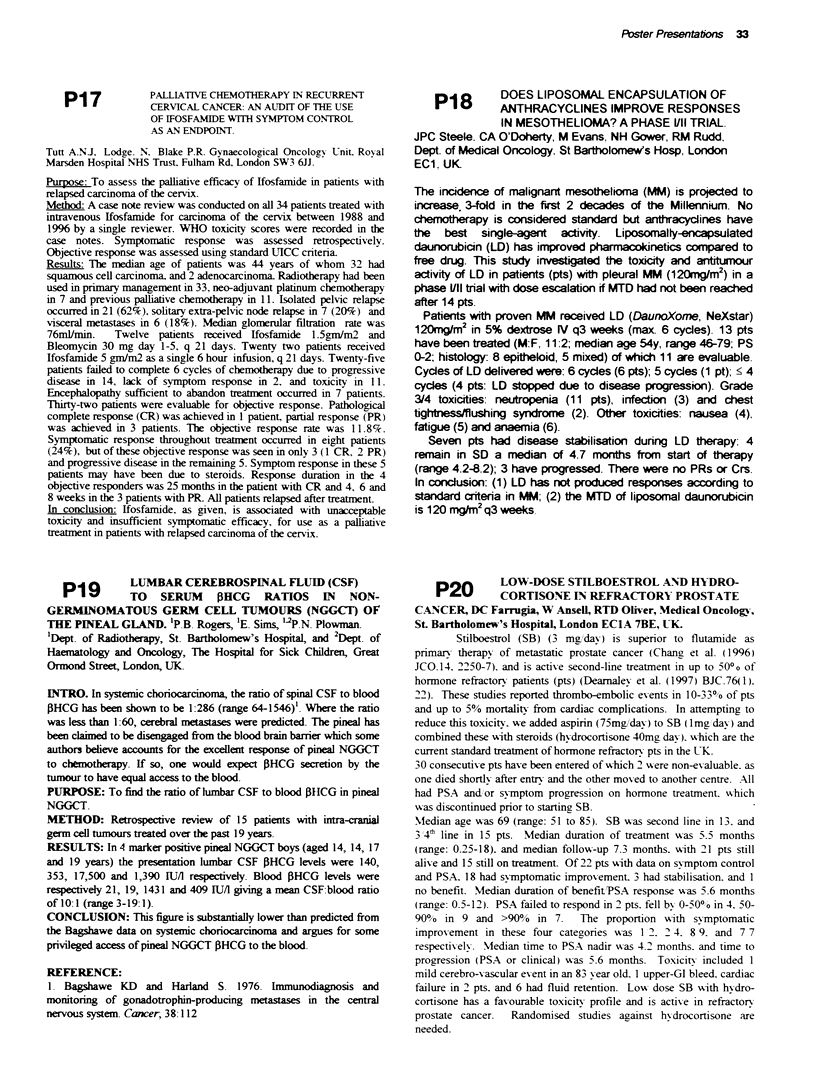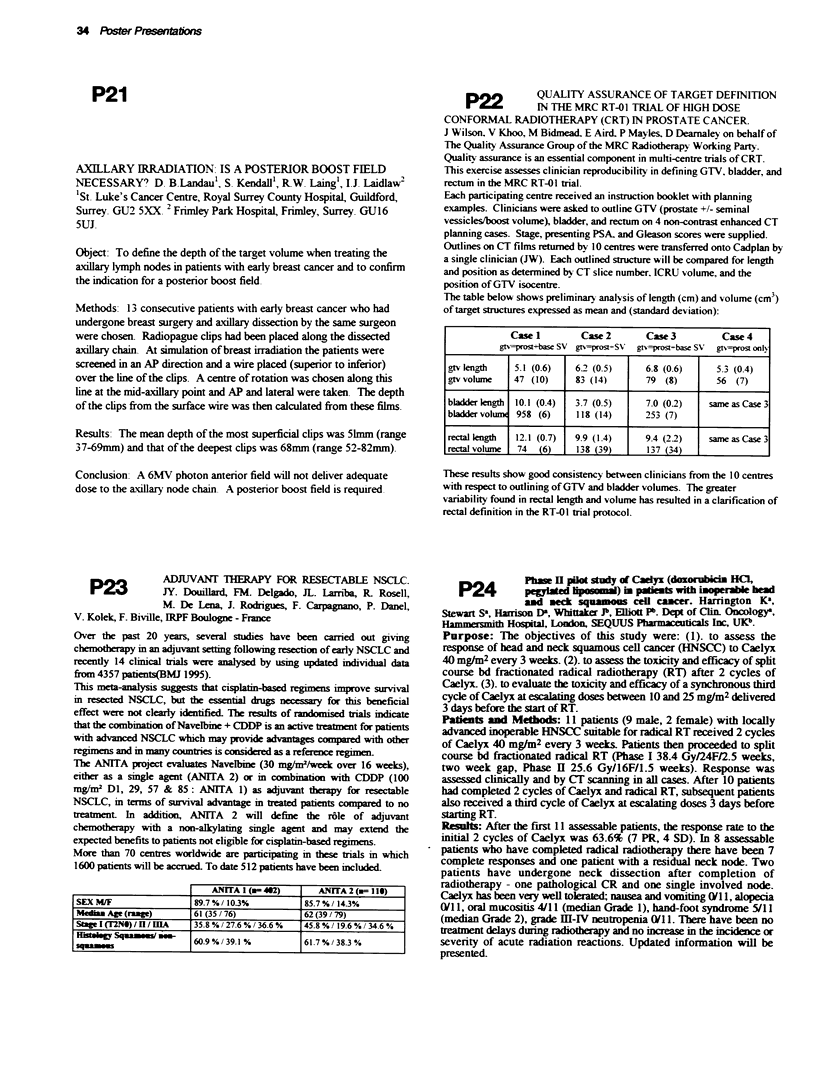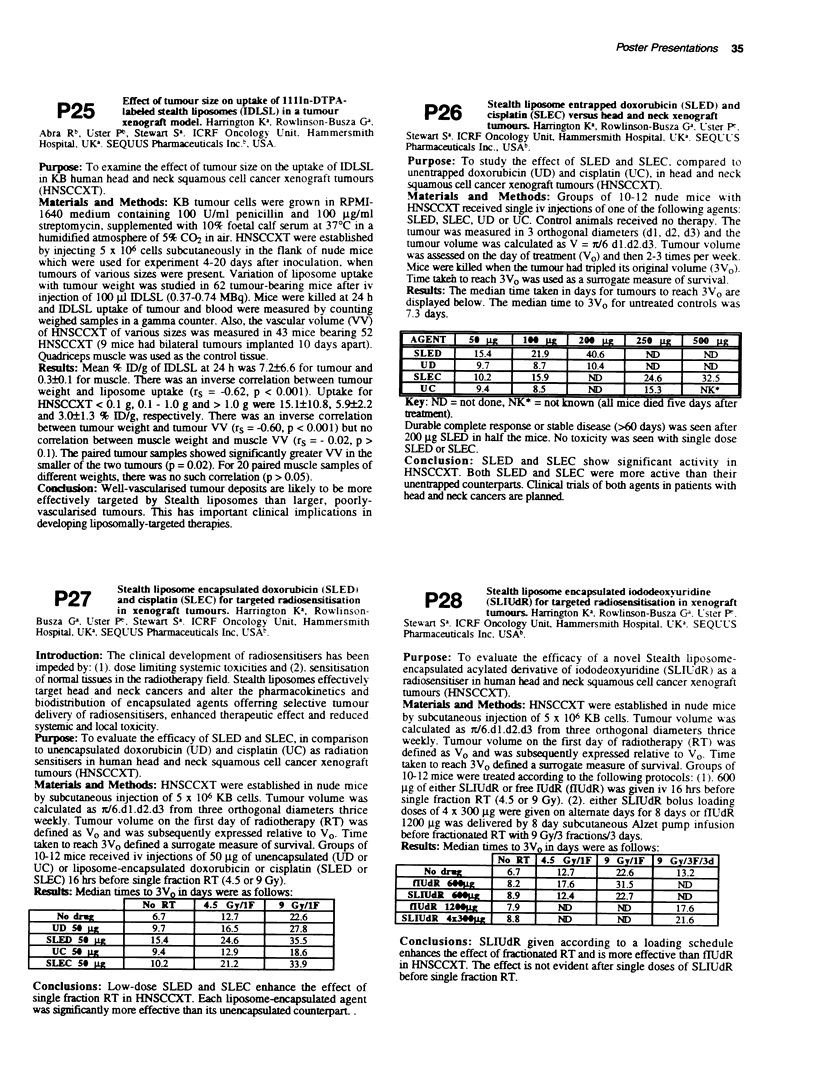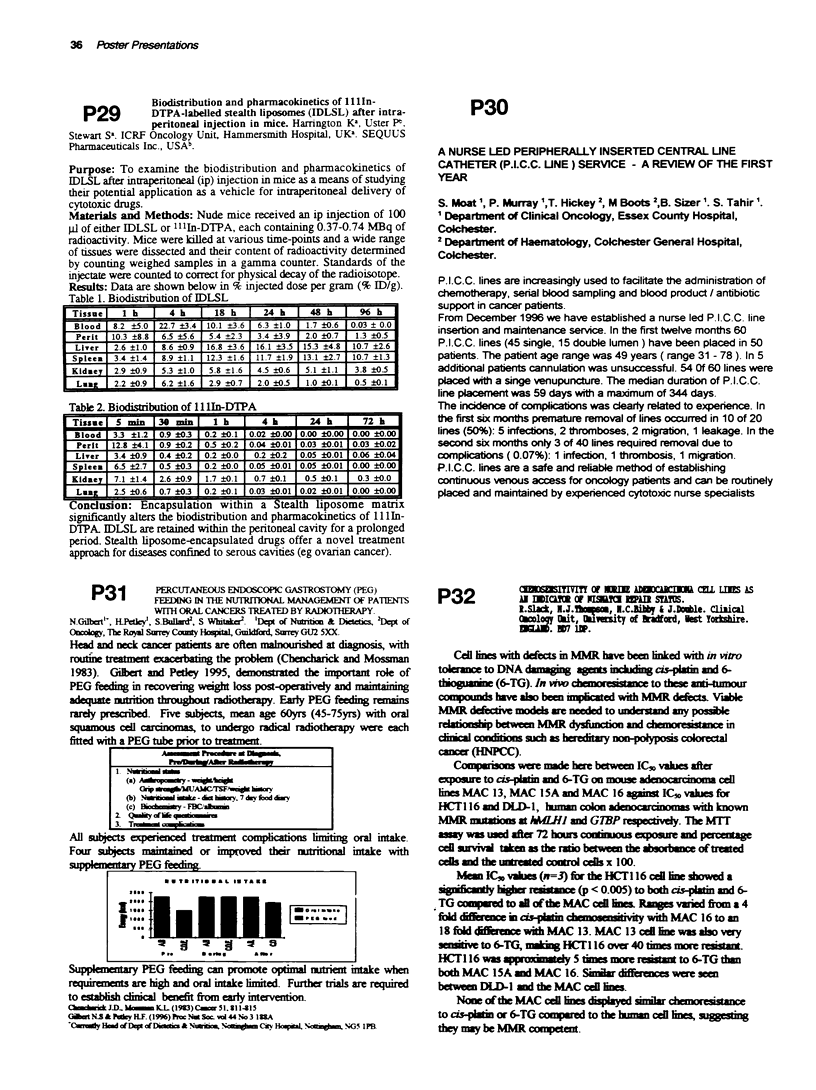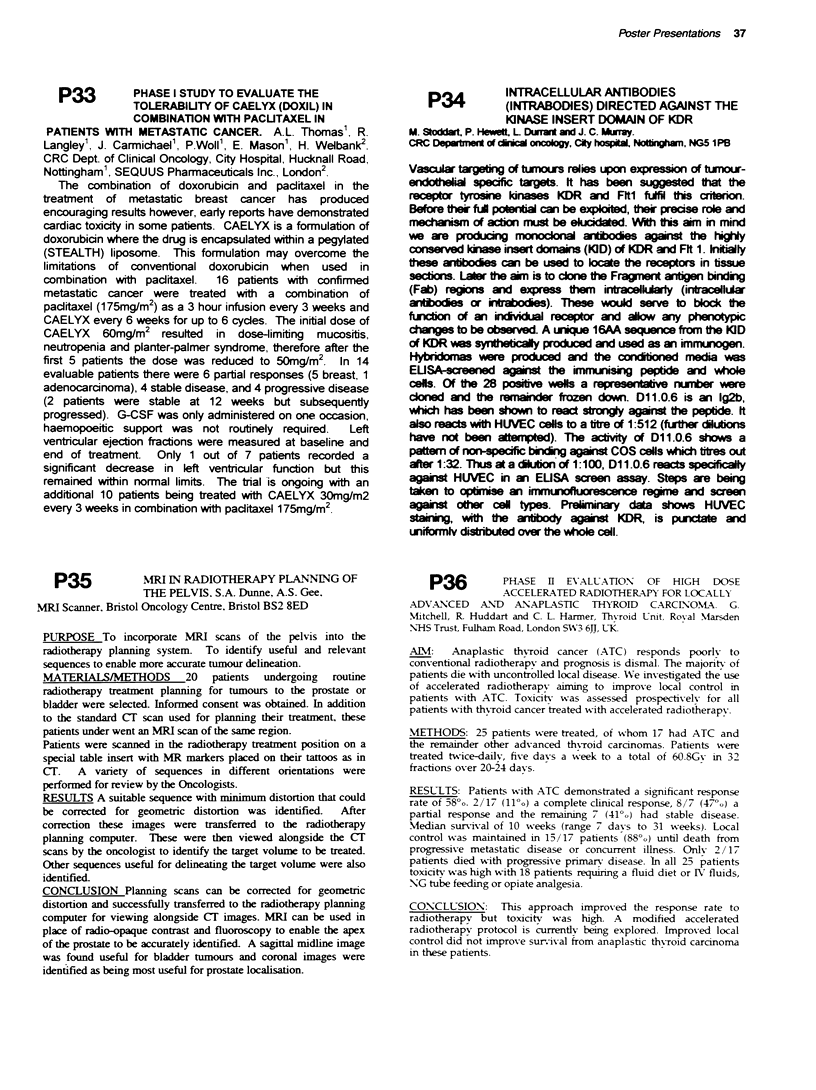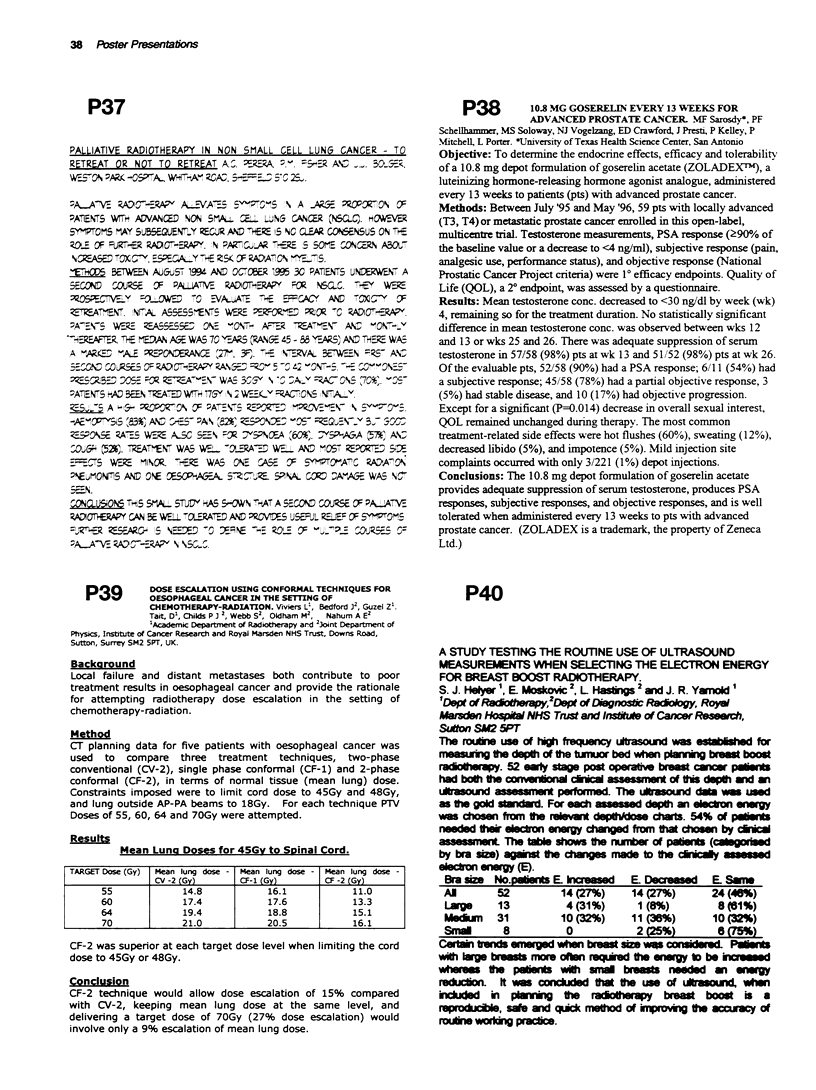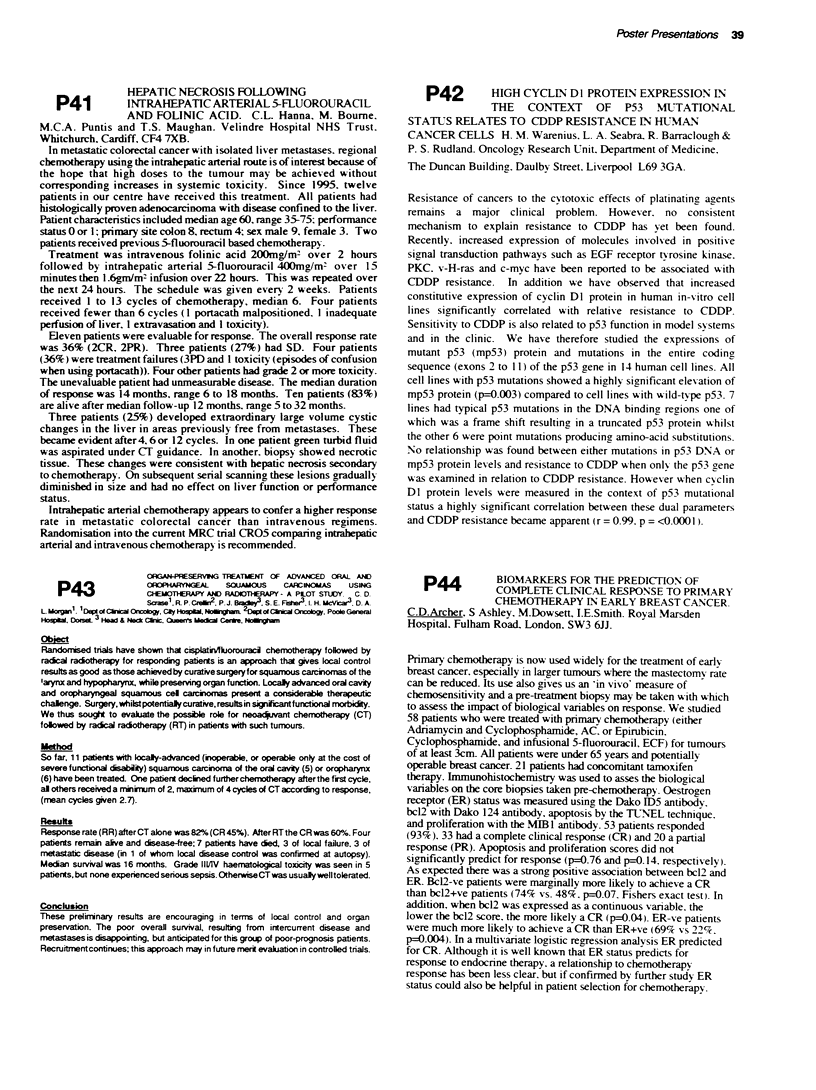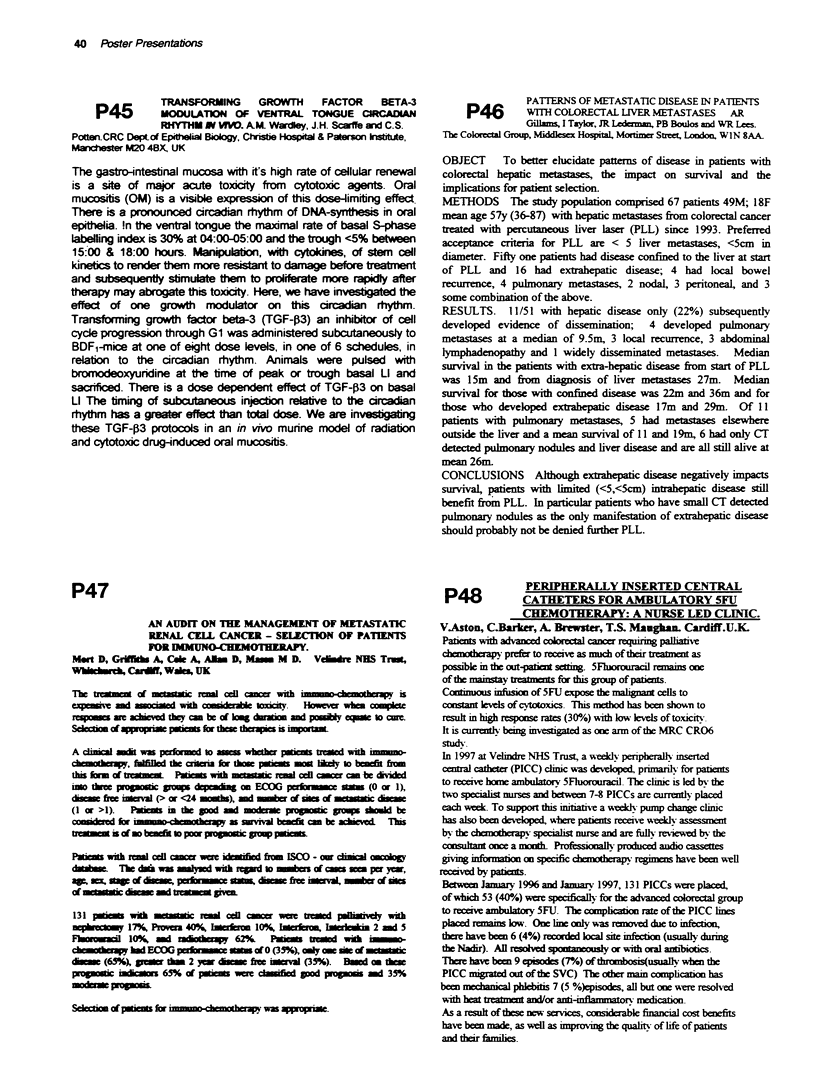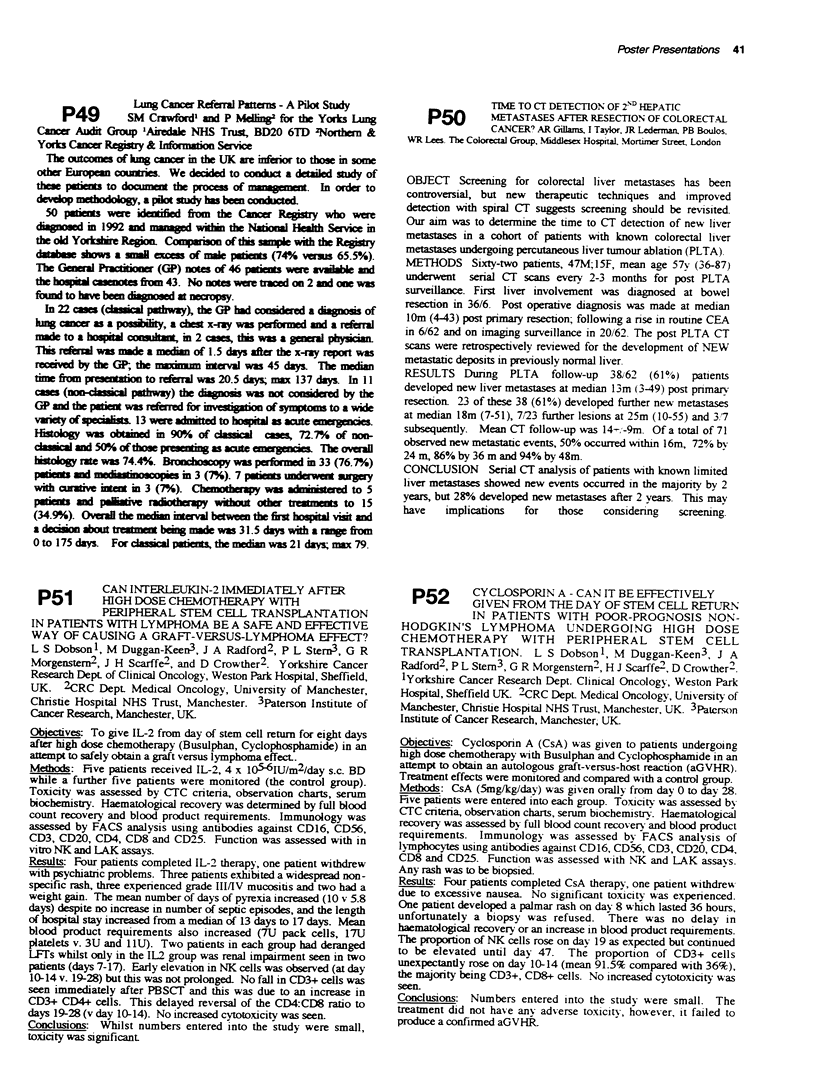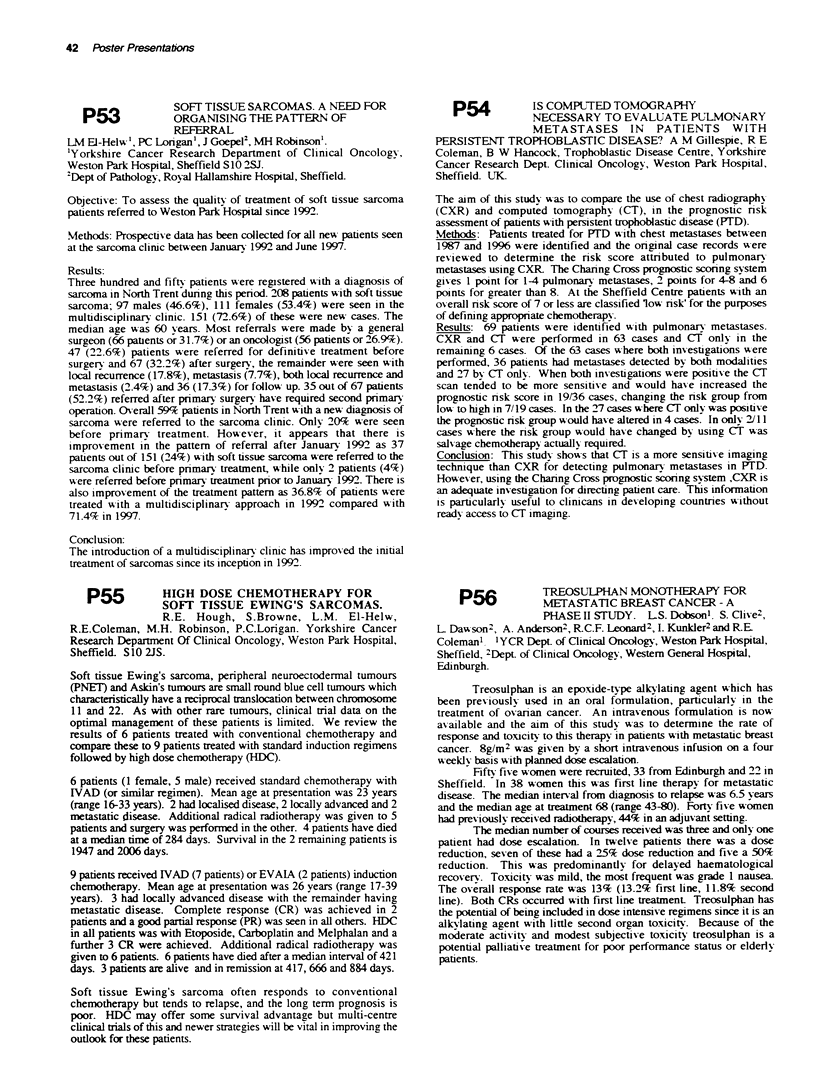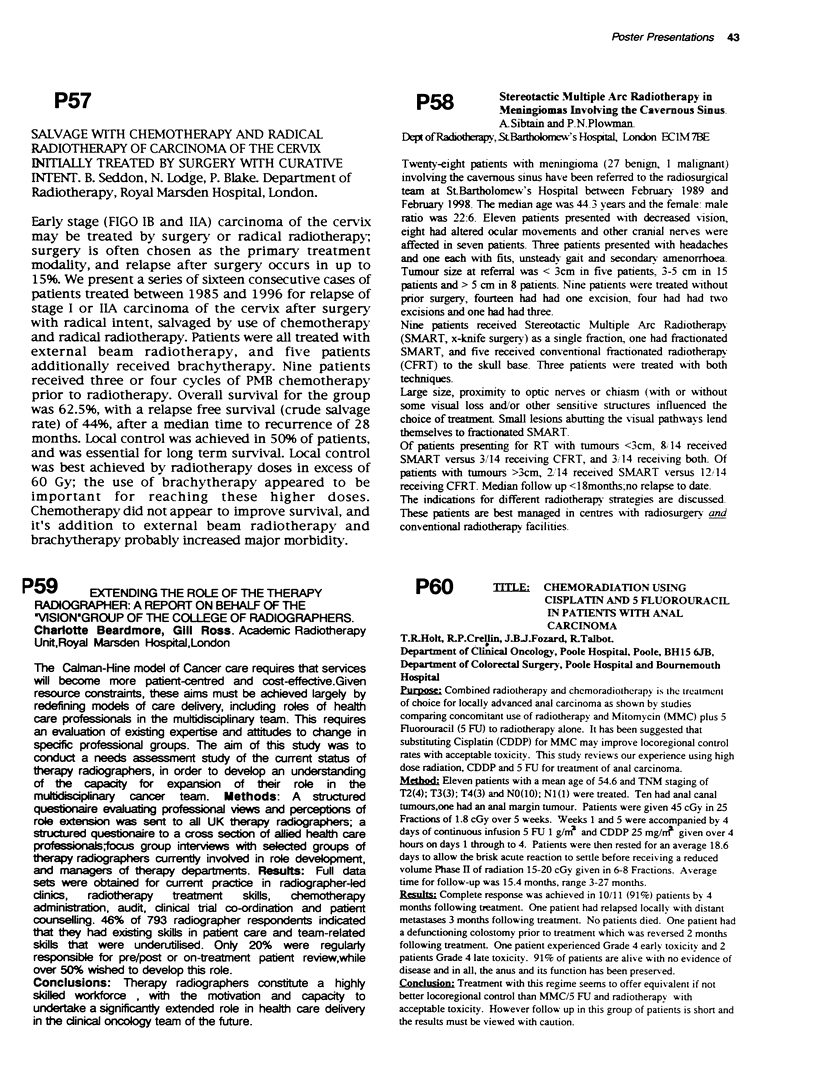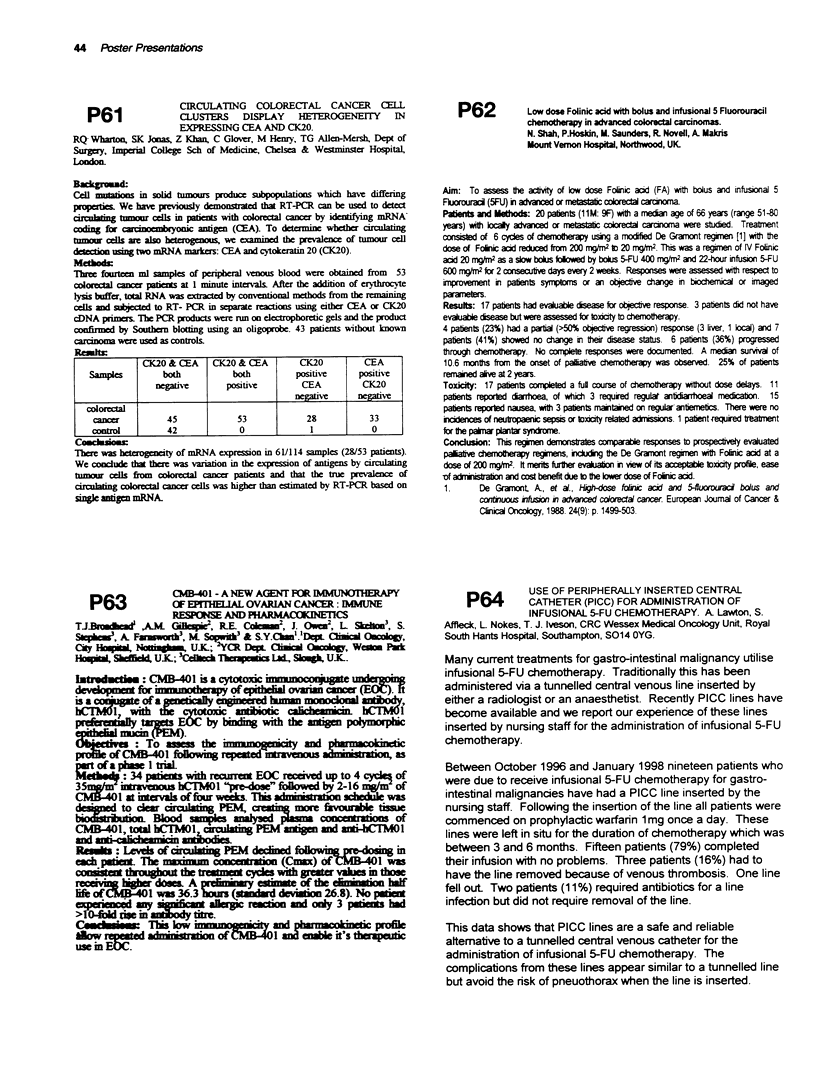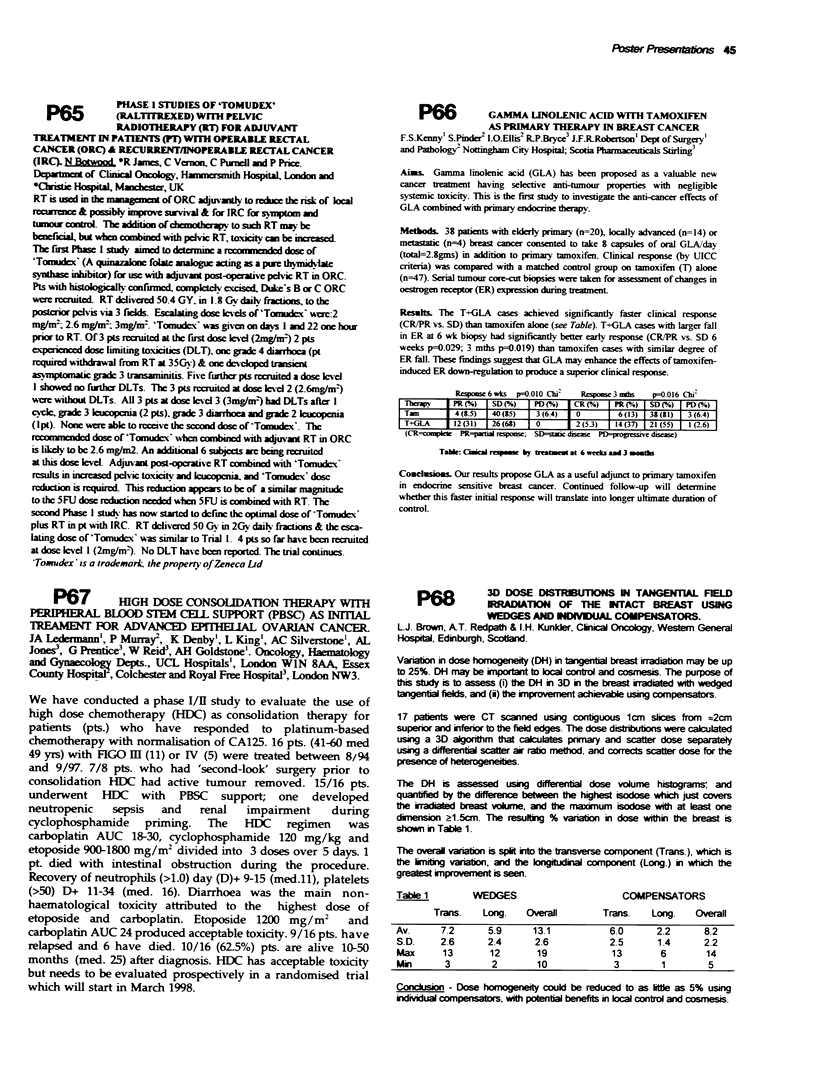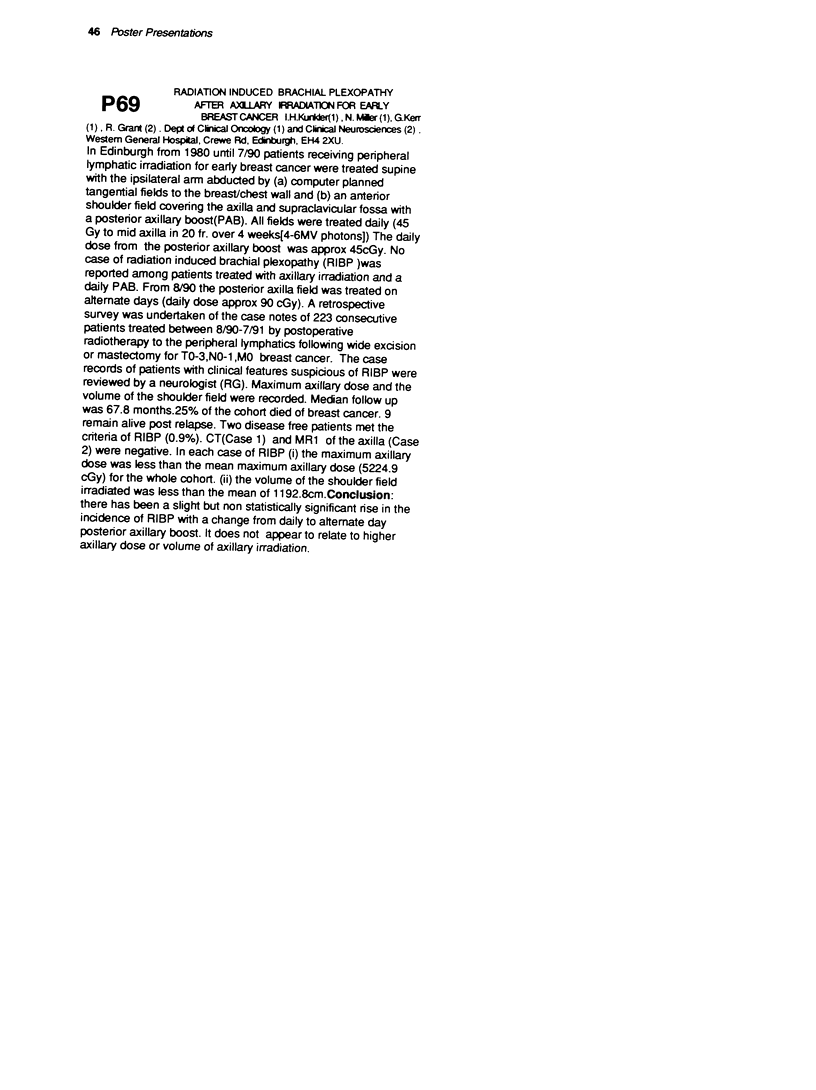# Poster presentations

**Published:** 1998

**Authors:** 


					
BnrTsh Joumal of Cancer f 1998) 78( Suppl 2 29-46
C 1998 Cancer Research Campaign

Poster presentations

Poster Presentatons 29

P01          RESULTS AND TF ADJUVNN AND

PAUAnVE       C1EIvKJHERAPY      FR     COLO-
RECrAL CANCER (CRC)NELDRYPAT
R.A. Popescu, A. Norman, P.J. Ross and D. Cnningham

The Depatment of Medicine and Gastrointestinal Unit, lTe Royal
Marsden Hospital, Sutton, Surrey SM2 5PT

Surgical treatment of CRC in elderly patients (aged 70 years or older)
has improved, but this growing populaon remains under-represented in
chemotherapy trials, with data on tolerability and benefits of cytotoxic
treatment still scarce.

Using   a  prospective  database  we   compared   demograpldcs,
chemotherapy toxicity, response, DFS, OS and QoL in 186 elderly
(median 73 years) vs. 658 younger patients (median 58 years) recevng
various 5-FU coaining regimens or Raltite for metastatic CRC.
Demographics (except age) and allocation to each regimen were similar.
No difference in response (24% vs. 29%, p=O. 19), median FFS (164 vs.
168 days) and 1-year FFS (18%h vs. 19%) was detected; median OS was
292 vs. 350 days (p=0.04) and 1-year suvival 44% vs. 48%1h. Frequency
of CTC m-Iv toxicity was similar.

124 elderly (median 74 years) and 419 younger patients (median 59
years) received adjuvant chemotherapy with protracted venous 5-FU or
5-FU/FA injections. A similar incidence of side-effects mandating a dose
adjustment (CTC grade II or worse) and severe toxicity (CTC m-IV)
was noted in both age groups, except for worse stomatitis in older
patients. In-patient stay was identical.

We conclude chemotherapy for elderly CRC patients is well tolerated
and has paliative benefits comparable to younger patients.

ADDITIVEEFFECT OF QUERCEllN COMBINED
P03         W1TH CARBOPLATIN: IN VITRO

CYTCYTOXlIaCYAND P53 INDUCTION. D. Fyfe,
A. Eliopoulos, C. Christodoulou and DJ. Kerr. CRC Institute for
Cancer Studies, Queen BiizabethHospital, Birmim  U.K.

ltoduction: Queretin is a naurally occurring flavonoid which
inhibits key enzymes within signal tranducio  pathways. A recent
phase I clinical trial quercetin combined with carboplatin showed that
the two can be safely given in combinatio: myelosuppression was the
dose limiting toxicity. We performed in vitro studies to see if there
was evidence of syner  in cytooxicityassays and in p53 induction.

Methods The in vitro cytotoxicity of qercetin, carboplatin, and
combinations thereof was assessed in the ovarian cnoma cell lies
A2780 and A27WJCP, using the M'T assay. Quercetin was dissolved
in DNO. CYtotoxicity was assessed after 72 hours exposure to both
drugs.

p53 levels in A2780 cells were assessd after 16 hours exposure to
quacetin  and   c       n   using  SDS-PAGE     followed  by

umumg mwith the monocdonal antibody PAbla)1 .

Results:  fDf    were calculated for a range of combinations of
queretin and carbolain and plotted on an isobologram. This
indictedan additive effect The EDso for quarcetin was 37?3.5 imnd
for A2780 and 32.5+?4.4 pamd for A2780CP cells. Cytotoxicity was
minima after 4 hours exposure only. DMSO delayed cel growth to
some       but also decased sensitivity to         by up to 5
fold.

Western bltting for p53 protein showed upregulation of p53 by
quercetin at 16 houms this increase in p53 was in adition to any
incre cawused bY cauboplatin aloe. 50 jiM quarcetin or 100 jM
auboplatin induced a 1.7 and 2.6 fold increase in p53 levels
respectively: combined treatment with the above drug c   s
increaed p53 expression 4.4 fold.

Conclui.n: Quefcetin and carboplatin have an additive effect in
cytotoxicity assays and induction of p53 levels in ovarian carinma
cell lines in vitro.

P02        OXAUPLATIN IN THE SECOND-LINE TREATMENT OF

FLUOROURACIL-RESISTANT COLORECTAL CANCER.

G Mlson', D Papamrchaef, JT Dente, F Richards', ML Slevin2 and
MT Seymour. 'ICRF Cancer Medicine Research Unit, Cookridge
Hospital, Leeds, 2St Bartholomew's Hospital, London.

Background: The novel platinum agent oxaliplatin has been re-
ported to produce a high response rate when added to 5-fluorouracil
and folink acid (5FU/FA) in patients (pts) with disease progression on
these drugs alone (EurJ Cancer 33:214, 97). Since March '97, we have
offered oxaliplatin on a named-pient compassionate use pro-
gramme (from Debiopharm SA, Lausanne) to selected pts with 5FU-
resistant disease in two British institutions. Patients: 20 pts with me-
tastatic colorectal cancer have been treated. All had evaluable dis-
ease; 60% had PS of 0-1; median age was 52. Sites of disease: liver
90%; looregional 30%; peritoneal/distant sites 70%. All 20 pts had
received previous 5FU: 2 had had only adjuvant therapy (Mayo regi-
men); 10 had received only one prior palliative regimen; 8 had re-
ceived 2 prior 5FU regimens. 16 (80%) had received infusional 5FU,
either as the 'deGramonr regimen or PVI-5FU with mitomycin. 18 pts
had well-documented disease progression during the prior 5FU regi-
men. Methods: Oxalipatin was given at 85mg/M2 as a 2-hour infu-
sion every 2 weeks. This was accompanied by the standard de
Gramont 5FUIFA regimen in 12 pts, by a modified de Gramont regi-
men in 7 pts, and by PV1- 5FU in 1 pt. Results: To date, 13 pts are
evaluable for response, with partil response in (15%), minor re-
sponse in 3 (23%), stable disease in 4 (31%) and progressive dis-
ease in 4 (31%). Overall, oxaliplatin has been stopped due to pro-
gressive disease in 5 pts and due to toxicity in 2, with 13 pts still on
treatment. In general, the addition of oxaliplatin to 5FU/FA is well tol-
erated: 4/20 pts have deveioped CTC Grade 3 toxicity, 2 myelotoxic-
ity and 2 peripheral sensory neuropathy. Abpecia, -nephrotoxicity
and ototoxicity were not seen. Conclusion: Oxaliplatin in combina-
tion with 5FUIFA is a useful and well-tolerated second-line treatment,
providing a further period of disease control in selected patients with
5FU-resistant metastatic colorectal cancer.

MONITORING OF STEROIDS
P04                IN A HOSPICE

J.White,A-Parr,S.Gomm

St Ann's Hospice, Manchester,U K.

Objective:To improve monitoring of and patient education re steroids.
Design:A policy for steroid use and a steroid record sheet were

developed -These forms were then reviewed with the case notes.

Subjects:Patients admitted during a 67 day period whom were on or
commenced on steroids.

Results:34/81(42r/) admissions were on steroids during the study period
92%! on steroids on admission 24/34(71%) were steroid record sheets
completed :58%! male and 42% female. Primary malignancy 35%

lung,13% for breast, brain and unknown pfimary.95% had meastatc
disease. In 33%/O a  i         steroid use was present 58 %
received G.I. protection according to protocol- 60 % given H2

antagoIMsts. 70%!. received dexamehasone, 25% prdnisolone and 5 %
both Duratin of Iratment r    8 % less than I week to 46%, for 5 to
52 weeks. Indiations- raised intracanial pressure at 38%I, appetite 21%
and well-being 25%.The commonest side effect was fingal infection

17/o.A clinical rsponse was observed in 83% of those on prednisolone
and 67/% of those tated with dexamethasone. During the study 75% of
the subjects died. Patient education by the hospice pharmacist was
performed in 33% of cases. All provisional discharge Letters were
completed fully and within deadlines of our policy. Of the final

discharge letters available 25% were completed for the GJ? within the

deadline of I week, however all had inrmton re steroid use outlined
in our policy. Referal forms lacked detail of steroids 7'!. no dose

recorded, 47/o had no response indicated and 27'/o had side effects
documented-

Coudcsions:Our method of using steroid record sheets and patient

education as tools to monitor the management of patients ta   steroidls

are effective. These methods are  appropatefor many hospice patients.
We ti    xem     end tie     m     in a selected pmnxizon of

hospice paients who have a rnable life expectancy

30 Poster Presentabons

P05

HEALTH ECONOMICS ANALYSIS OF HOME BASED VS. HOSPITAL BASED
CHEMUIXERAPY: INTERIM RESULTS
IngLeby. S. Baker. J.2. Young. A.1. Kerr, DJ.

CRC Institute for Cancer Snudies. Birmngham Univerity. 2University Hospital Trus.
Birmigham, UK

I          : The current climae within cancer fanagenent in the UK. follwing the
'Cama/HE'                 (1 994), is detailed evaluaion of the merits of patient
ueatment opooincluchng cost effectveness.

In Biinngham. a feasibiity and economic analysis of home based chemotapy for

adNlanced colofectal cancer is currently running as a sub-study of the MRC CR06 Trial
(1 997). using the Tomadex, DeGramont and Lokich regins.

Method: Patients are assessed and tated at home for the first 12 weeks of treatment.
by a designated research oncology nurse. A Hickman or P1CC line was inserted for
Lokich and De Gramont Regimens. Costng of home based care was based on the
nurse's hourly rate for time, trael. and phone calls. Standard hospital charges for

hospitalisaton or clinic visits were used as the compaison. AsSInIons made were

that the pottial risk of coiplcatons and toxicity for both ams (bome and hospital)
m the 3 regmens was the same.

R   ks: first 7 patients conaleting 12 weeks of tratment (mean values):

Tomdexn=2          DeGramnit n=2      Lokichn=3

Hospital based Tomadex  = ? 368.00  V. Home based Tomudex  = ? 261.00
Hospital based De Gramont = ?3036.00 V. Home based De Gramont = ?157930
Hospital based Lkch    = ?1104.00 V. Home based Lokich   = ? 855.92
co~~o. Tbesc early costng resuls of a small sample of aents indicate that

Home Chemotherapy is cheaper per regimen than hospital based ratment. The stdh,
will continue, to obtain sample size of 51 for significant results.

Rdem: Dept of Health, 1994). Cosulaive           A PoLb Frtlc for
CmusoCancer Srie,Loodon.

MRC ( 1997) CR06: Clinil ProtocoL Feb. MRC Cancer Trials Office, Canbndge.

P07                 PHASE lB STUDY OF CONCURRENT

ADMIIMSTRATION OF MARIMASTAT

AND GEMCITABINE IN NONRESECTABLE
PANCREATIC CANCER

J.D.White (1), J.Carmichael (l),P.J.Woll (1), T.Guilford (2),

R.C.Russell (2) ,(l)Nottingham City Hospital, Nottingham, UK, (2)
University College London Hospitals, London, UK.

Aims: 1 to assess safety and tolerability of a range of marimastat doses
when concurrently administered with gemcitabine in patients with
non-resectable pancreatic carcinoma. 2 to assess the effect of this
combination on progression of pancreatic carcinoma.

Design: a dose escalation, phase I b study Patients received
gemcitabine 1000 mg/M2 every 3 weeks with a one week rest period
(cyclel) repeated to a maximum of 6 cycles The marimastat dose was
increased in each cohort from 5 mg bd.. to 20 mg bd. in 5 mg
increments Progression was evaluated by CT scan, CAl9-9 and
clinically. Toxicity was assessed using clinical examination and
laboratory parameters. Subjects: 31 patients (17 male, 14 female) with
non resectable pancreatic cancer were recruited from 2 centres between
September 1996 and September 1997. Eligibility cnrteria included an
ECOG performance status of 2 or more and no prior chemotherapy or
radiotherapy.

Results: 10 patients completed 6 cycles, 12 were withdrawn due to
disease progression or patient refusal after mean of 2 cycles ( range 1-
5).Nine patients are still ongoing (mean 3 cycles). Overall treatment
time was 109 months. Six patients reported musculoskeletal problems-3
stopping and 2 reducing marimastat. Gemcitabine was delayed or dose
reduced in 5 cases. Toxicity contributed to witdlrawal of 2 additional
patients. Biochemical toxicity - 1 grade 4 raised bilirubin and 4 grade 3
deranged liver function tests. Nine grade 3 myelosuppression. Two
grade 3 back pains. Early response data according to radiological
criteria in 11 assessable patients. 2 responses and 6 stable disease.
CA19-9 levels fell in 9 cases.

Condusion: the combination of gemcitabine and marimastat is well

tolerated and merits fiuther trials for dose setting and scheduling

P06          A MODIFIED "DE GRAMONT" REGIMEN FOR

USE IN THE CHEMOTHERAPY DAY UNIT

MT Seymour G WAlson. JT Dent, S Fa/lon & F Richards. ICRF Can-
cer Medicine Research Unit, Cookridge Hospital, Leeds LS16 6QB.

Backaround: De Gramont's LV5FU2' regimnen - FA+FU bolusfmfus-
ion for 2 days each fcrtnight - was superior in a phase III tria to stan-
dard ayo' 5-day bolus FU/FA as first-line treatment for advanced
coorectal cancer (J Ciin Oncol 15:808, 1997). It has been used in sev-
eral MRC tbds and is widely used throughout Europe. But it is expen-
sive bth of resources and of pabets' time: the high dose of FA (400
mg/mrkyc1e) is of dubious contrbution, and ft irmles daiy ouqSer

visAs with a pump system, or an ffpient stay. We have tror modi-
fied the reginen to nake it sfnpIer, cheaper and more converniet for
outpetient pracice. MNhods: 29 pts with advanced GI trad cancer
have been entered. 63% are male; median age 65 (range 49-78). PS = 0
in 41%, 1 in 21%, 2-3 in 38%. Pfimy site: 17 cordal, 7 gasic, 5
awer. 34% had received pnor chemo. Pts were fitted wih subcut. wenous
injecion ports and anticoagu"ed to INR 1.4-2.0. Treated was adminis-
tered on the day wd using porabe punps. Tobxiciy1PS assessmnt
was suppemernted by QLQ-C30 questonnaies. The regimnn: FA 350
mg (ficed dose) over 2 hr, then FU 400 mg/Tn2 5-mi bolus, then FU 46-
hour iffusion at escaliang statbng doses of 2000 mg/rn2 (level #1), 2400
(#2), 2800 (#3) and 3200 (#4) mgmh2 for successive chorts, with wihin-
pafient dose escaietion, to estabish the mtaxmum toerable dose (MTD).
Resulb: The regimen is convenient and generqy wel tolerated. Dose
reducions, if made, are most often for mild but persistent nausea, leth-
argy or diarrhoea Treetment was toerWaed withot dose reduction by
5/6 pts at level 1, 9/10 pts at level 2 and 9/12 pts at lvel 3. Level 4 was
tolerated by 7/9 pafients, but 2 pts had CTC grade 4 toxicy (1 CNS; 1
worrmit) which was nd seen at br levels. Tnrernt is ongoing and
at this point only 14 pafients are assessable for response, with 5 PR.
Conclusions: At level #3, the new reginen, despite Ks 60% higher 5FU
dose itensity, has simar toxicity to standard -de Garnon LV5FU2, but
is more cornvenient, less expensive and could pdentialy be considred
in the adjuant sefting. A randoffse comparson would be required to
conffim equivalent efficacy.

P08           THYMIDYLATE SYNTHASE (TS) AND P53

PROTEIN     EXPRESSION      IN   PATIENTS
WITH RECTAL CANCER TREATED WITH

PRE-OPERATIVE 5-FLUOROURACIL (5-FU) BASED
CHEMO-RADIOTHERAPY (CRT). S.A. Ward', S.P. Joel', D.
Papamichael', M.L. Slevin', R. Glynne-Jones2. 'Department of
Medical Oncology, St Bartholomew's Hospital, London, 2Mount
Vemon Hospital, Northwood, UK.

Purpose: Pre-operative 5-FU based CRT has been reported to be
beneficial in patients (pts) with rectal cancer. Expression of TS protein
(as a target of 5-FU) and/or p53 protein have been shown to correlate
with the outcome of 5-FU based treatment in advanced colorectal
cancer. In this study TS and p53 have been quantified in pts receiving
pre-operative CRT. Methods: 30 pts with advanced/fixed rectal
cancer received pre-operative CRT. Radiotherapy was 45 Gy in 25
fractions (3/4 plan field), and chemotherapy was folinic acid 20mg/m2
iv bolus followed by 5-FU 350mg/m7 as a 30-60 minute infusion on
days 1-5 and 29-33. Clinical response was evaluated 4-6 weeks after
comple-ion of CRT by standard UICC criteria; complete response
(CR), partial response (PR), no-response (NR). Pre-treatment paraffin
tumour sections were stained immunohistochemically (IHC) for TS
using a polyclonal antibody and p53 using the D07 antibody, with a
streptavidin-HRP method, and scored for intensity (0-3+) and extent
of staining. Results: The IHC data were analysed in relation to
clinical response, as shown in the table.

p53,,.    p53+,,    TSL.     TSw.
NR           3         3         3         3
PR/CR        16    |   8         13       1 1
j Total            30                 3 O

There was also a trend toward normal (-ve) p53 and low TS score
(data not shown). Conclusions: Tbese data suggest an association
between normal (-ve) p53 and response, but not between TS and
response, although currently the number of observations, particularly
in the non-response group, are too small for these trends to reach
statistical significance. Data on an additional 50 patients will be

presented.

Poster Presentabons 31

P09

ATTITUDES TO CHEMOTHERAPY IN THE OLDER PATIENT:

RESULTS OF A QUESTIONNAIRE SURVEY

P Murray, S Moat, B Sizer, S Tahir

Department of Clinical Oncology, Essex County Hospital, Colchester

A questionnaire survey of cancer patients, health care professionals
and matched controls demonstrated that patients with cancer are
willing to accept intensive chemotherapy treatment with a very small
chance of benefit compared to the other groups studied. (Slevin ML
et.al. BMJ 300 (6737): 1458-60).

Advances in chemotherapy and the management of side effects are
making it increasingly feasible to treat older patients with both radical
and palliative intent.

To examine whether older patients are prepared to accept such
treatment, we have applied the previously published questionnaire to
60 cancer patients over 70 (range 70-84) about to commence cytotoxic
chemotherapy.

The median benefit required to make hypothetical intensive treatment
worthwhile for a chance of cure was 50%   (range 1-100%), for
prolongation of life was 2 years (range 3 months to 5 years) and for
symptom relief was 50% (range 1-100%).

For chemotherapy causing mild side effects the median benefit
expected for a chance of cure was 25%, for prolongation of life: 2
years, and 25% for relief of symptoms.

These data imply that older patients' attitudes to chemotherapy might
differ significantly from  younger patients.  These differences in
expectations and preferences need to be considered when
recommending treatment to older patients with cancer.

P1I1 CAFFEINE POTENTlATES CELL DEATH IN p53
MUTATED RADIORESISITANT BURKITT'S LYMPHOMA
CELLS BY APOfTOSIS AND INCREASING THE
FORMATION OF GIANT CELLS. T. M. Illidgel; J.Erenpreisa2;
MiS.Cragg' and M J. Glennie'. 1. Lymphoma Research Laboratory,
Southampton General Hospital, Southampton S016 6YD.
2-AKirchenstein Institute, Riga LV-1067, Latvia.

In this study we have investigated the role of caffeine in abrogating
G2 cell cycle arrest and increasing apoptosis in p53 mutated Burkitt's
lymphoma cells following single high dose irradiation. After

genotoxic damage a cell must communicate signals to both DNA
synthesis and mitotic machineries in order that a mitotic block is not
followed by an extra S phase, or vice versa- In the absence of
fimctional p53, cells arrest in a G2 like state after irradiation in a dose
dependent manner. When this protracted G2M arrest is overcome,
addiional rounds of DNA synthes and abeant mitoses follow as
part of an endocycle. The balance between the DNA syndtesis and
nuclear divisions favours increasing of the DNA content, with cells
acqwrng increasingly gigantic polyploid nucli, upto 128N DNA
content. Caffeine increased (2-2.5 fold) the level of early apoptosis,
72 hours after treatment and caused a 2-fold increase in both the
nunber of polploid cells and an increase in the mean polyploidity.
Caffeine appears to enhance the formation of giant cells, which

sbsequently undergo multinuclear apoptosis. This disintegration of
giant cells reaches an apoptotic crisis around day 5-7 and is the
predominant mechanism of p53-independent apoptosis. In contrast to
recent reports suggesting that abrogation of G2 arrest by caffeine

icreases apoptosis in p53 inactivated tumours; we demonstrate that
this does not appear to be the most important mechanism in Burkitt's
lymphoma cells. Instead the formation of giant cells and inhibition of
nucleotide excision repair may be more important mechanisms.

(This work was supported by the Cancer Research Campaign)

Pi0         NON-HODGKIN'S LYMPHOMA

PRESENTING WITH SPINAL CORD

COMPRESSION. AC McDonald*l, JAR Nicoll2 and RP Ramplingl.
lBeatson Oncology Centre, Glasgow GIl and 2Dept of

Neuropathology, Institute of Neurological Sciences, Glasgow G51

Between 1985 and 1996, 25 patients (lIM, 14F; median age = 62,
range = 16 - 78yrs) presented with spinal cord compression due to
previously undiagnosed non-Hodgkin's lymphoma (NHL). At
presentation 17pts were non-ambulatory, with bladder dysfunction
observed in 8pts and dual sphincter impairment in a further 9pts. The
thoracic spine was the commonest site of disease (17pts) with other
areas less frequently affected (cervical = 3; lumbar = 4; sacral = 1).
Twenty pts had intermediate or high grade tumours (stage I = I0pts;
stage  1 = 1; stage  1 = 2; stage IV = 7); l1pts were treated radically
using combination chemotherapy (CT), with 5pts receiving
consolidation radiotherapy (RT). Two pts relapsed (without local
failure) at 30 & 53 months and received further CT. Five pts, of initial
poor performance status, with stage I disease received radical RT
alone, of which lpt has relapsed Four pts were treated palliatively.
Overall llpts of this group remain alive and disease-free after median
follow-up of 36 months (range 12-63). Three pts with stage I low
grade disease received radical RT, of which 2 have relapsed, both
without local failure. All 5 pts with low-grade NHL remain alive after
median follow up of 8.5yrs (range 20-133 months)

Overall survival at 5 yrs is 600/o, with initial ambulatory status and
faecal incontinence associated with survival (both p<0.02). The
majority of surviving pts became ambulatory by 6 months (17 of 18
surviving pts) with similar improvement in sphincter function, in
contrast to cases of SCC in metastatic carcinoma. This diagnosis
merits consideration in all pts with SCC attributed to malignancy.

P12           A FEASIBILITY  STUDY OF VINORELBINE (VNR)

AND GEMCITABINE (GEMf) IN INOPERABLE
STAGE IIIB-IV NSCLC. K Mattson. K Tamnimen,
V. Nlkkanen, M. Vi  OP. Isokangas, FM. Delgado, I. Barbet, Helsinki-Finland
Objective: VNR and GEM have significant activity in NSCLC. both as
single agents (RR = 21-30 %) and in combination with cisplatin (RR = 30-
40 %). This phase I-II trial aims to evaluate the response to concurrent VNR
and GEM, as well as response duration, time to progression, survival and
toxicity.

Patients and methods: Patients initially received VNR 30 mg M2 week on
days 1, 8, 15 and 22 of a 28 day cycle, and GEM 1000 Migm2 on days 1. 8
and 15. Initial treatment consisted of 3 cycles with pts achieVing NC PR CR
being given a further 3 courses, and those with PR, CR after
6 courses continuing the treatment until relapse. Entry critenra include
histologically or cytologically confirmed Stage EIIb or IV NSCLC. no
preVious chemotherapy or radiotherapy, PS ? 2.

Resuts: Neutroxema has necessitated reductions or delays in the
administration of VNR and/or GEM in the first 7 patients. We have therefore
modified the schedule to VNR 30-35 mg/ml and GEM 1000-1200 mg/T2 on
days 1 and 15 of each 28 day cycle. This fortnightly schedule allows the full
protocol dose to be administered as well as being very convenient and cost-
effective. From the previous analysis. no G4 Neutropenia was observed and
only 6.5 % G3 per cycle has been recorded so far.

Conclusion: the phase II is on-going and 27 out of 30 planned pts have been
enrolled. Preliminary results will be presented.

32   Post Presentatons

P13           A PHASE 1 STUDY OF XRO0 BY 3HR IV   FUSION.

J.d BoJ. ', NBlechan, D.Se3, PBe3, SMoor3,
J.ShadenJ-Dcmis, Ludson3, C.Twdves'. CRC Dept ofMed Olcot Glasgow';
MRC Dept. of alinial O ejog  lmudge2; Royal Marsd  H   SWT

XRSOOO (DACA), is an aidinc divative which inhits       I and
IL A pont cytoxic, it can ovrcome several n-chiks of muhidrug
r   nce. In this study XR 5000 was gie as a 3 hr IV infu n n 3
suna ssiv days every 3 eks. 41  s (27 male14 femake median age: 55
yrs - range: 31 to 73 yrs) were tated at 11 do e ls ranging from 9 to
Mznghn7(dos lqp es  as salt). The  nncst and dose-niming toxicity was
infaim arm pim which was        and variabie in inensity at dose lvls
up to and inchling 600mgin2. Tbc arm pam was more  e   _
and do     g at 800mg/mr2 ajihoug reeri  with no  acb  One
reased through a cetral ven  cat  expened cst pam with

ECG chanes, but no mycal       o     At the highest dos lvis several

-       also cxp, imned flushing ohe asuciatd with pemal _aracsae
and a felng of a  i    Nausea, vomiting,      and a6ecia we

-          Only one  -    -eisgmciaDt myopeedsi       This was
an u...  licae and tansie isoe    f grlade 4              and
z~[4zropeia obscrved at the higlw dow level (8Omg/rn2) At this dose lvel 3 of
9   paies developed signifiact txcity  c ag withdrml of uteatnnt

00mg/mw2   a    is  d  as the maxim      toleraed dose. DACA

ecic  lina I aver the dos-ag Shxi Alhough I pcfen
with   astibreast   had a rerwmin ohmg nmcstase, dtre were no
oljet1ive respom s This paten of drug toxicity is mmsual and it may be posbe
to aminieiger dh      f XR5000 usin a difent -dmninistratir sheku .

P15           NEW AND IMROVED- THE UKCCCR RIEGISTE

OF CANE TS,

Gr.oss   , Brown P.C., Stewart L A., Fayers. PM.,
MRC Cancer Trials Office, Cambridge, CB2 28W.

OBJECTIVES: The UKCCCR Register of Cancer Trials is a register of UK
randomised controlled trials (RCT's) in cancer. Our main objectives are
to produce and maintain a comprehensive and up-to date register of
UK RCT's in cancer, to makle the UKCCCR Register as widely available
and easily accessible as possible, and ultmately to ensure that all trials
are prospectily registered with the UKCCCR Register.

METHODS: Following consulation with UKCCCR Register users and
the povdes of trial data a core dataset of trial information has been
established which indudes information on trial design, patient accrual,
main trial contacts and organising and funding bodies. Copies of trial
publications and protocols are kept on file.

The UKCCCR Register software has recently undergone considerable
re-deVelopment and indudes iMPrOved search and reporting facilities.
Search results can be viewed on screen, printed or downloaded to
disk. Users are able to select the level of detail they require.

RESULTS: In February 1998 the UKCCCR Register contained over 500
UK RCT's in cancer which had been registred, both pro ctively and
retrospely, by the apprpriate   searcher. More than a quarter of
the trials were open to patient accrual, and over 70 new trials had been
registered in the preceding six months.

The UKCCCR Register can be downloaded by collaborators from our
Web site at http//.www.mrc-cto.camac/ukcccr/, and is also available
on floppy disk (five disks) and CD-ROM for use in Microsoft Windows.

FUTURE Future areas of focus will be to collaborate with funding
bodies and ethics comittees to persuade them to encourage the
prospective registration of those trials under their remit, and the
development of a fully warchable version of the UKCCCR Register
which will be available over the Intemet

P14

DOES REFERRAL FOR TREATMENT OF
SPINAL CORD COMPRESSION OCCUR
MORE FREQUENTLY ON A FRIDAY
AFTERNOON?

RS.D Brown, S. Rajaram, PJ. Ostler, RJ. Burcombe and H.A.
Payne.   Meyerstein Institute Of Oncology, Middlesex Hospital,
London, WIN 8AA.

Anecdotally, emergency referal for treatment of spinal cord
compression often occur late on a Friday afternoon. To assess this we
performed a retspective analysis of 102 patients teated for spinal
cord compression (SCC) between April 1995 and October 1997 with
particular reference to time and date of treatment

MetxI Patients with SCC were identified from the simulator
planning records. Departmental machine log books were then
reviewed to obtain the time and date of each patient's first fraction of
emergency radioterapy.

Results: One hundred patients received their first emergency treatment
between Monday and Friday. Thirty-two patients were treated on a
Friday. Statistical analysis with a chi-sqaed goodneof-fit test
showed this to be significantly more common than any other
treatment day ( p < 0.05).

Sixty-seven patients were first treated after 4 pm. and 48 after 5 p.m.
We could not find any association to suggest that treatment on a
Friday was any more likely to be later in the afternoon than for other
weekdays.

Concluiion: Friday afternoon is the single most common teatment
ime for emergency SCC. This ocurs because Friday is significantly
commoner than any other weekday and not because a late afteroon
treatment time contributes this phenomenon.

P16

INTRAVENOUS ANTIEMETIC

TREATMENT WITH GRANISETRON 1 MG
OR 3 MG FOR CMF CHEMOTHERAPY.

RS.D Brown, PJ. Ostler, D. Hoare and M.N. Gaze. Meyerstin
Institute Of Oncology, Middlesex Hospital, London, WIN 8AA.

Three  weekly    intravenous  cyclophoshamide  600  mg/m2,
methotrexate 40  mg/n3 and   m-fluorou     600 ng/mn (CMF)
chemotherapy is widely used for treatment of breast cancer in the
United Kingdom. The 5-hydroxytrypmine (5-HT3) antagonist
granisetron is a standard antiemetic used in combination therapy to
prevent nausea and vomiting. European dose recommendations
suggest 40 mcg/kg (3 mg) but North American data sheets advise 10
mcg/kg (1 mg). We have compared the effectiveness of these 2 doses
in an anempt to define if the lower dose was equally effective. This
would allow a reduction in drug costs for antiemetic use.

MeUtxA Retrosective analysis of the case records of patients treated
between Februay 1995 and October 1997.

Rcu&ts: 49 patients with either granisetron 3 mg (n-22, 102 cycles of
CMF) or 1 mg (n=27, 123 cycles CMF) were compared. Emesis rates
for granisetron 1 mg were similar to 3 mg when expressed either as
per patient (9 % vs. 7 %) or per cycle of CMF (2 % vs. 3%). Nausea

rates for granisetron l mg were similar to 3 mg when expressed either
as per patient (55 % vs. 48 %) or per cycle of CMF (44 % vs. 33 %).

Conclusion- The rates of both nausea and vomiting was not
significantly different for granisetron I mg or 3 mg. This allows the
use of the lower granisetron dose with a sbstantial cost saving.

Poster Presentabons 33

P17

PALLIATIVE CHEMOTHERAPY IN RECURRENT
CERVICAL CANCER: AN AUDIT OF THE USE
OF IFOSFAMIDE WITH SYMPTOM CONTROL
AS AN ENDPOINT.

Tutt A.NJ. Lodge. N. Blake P.R. Gynaecological Oncology Unit. Roy al
Marsden Hospital NHS Trust Fulham Rd. London SW3 6JJ.

Pupose: To assess the palliative efficacy of Ifosfamide in patients with
relapsed carcinoma of the cervix.

Method: A case note review was conducted on all 34 patients treated with
intravenous Ifosfamide for carcinoma of the cervix between 1988 and
1996 by a single reviewer. WHO toxicity scores were recorded in the
case notes. Symptomatic response was assessed retrospectively.
Objective response was assessed using standard UICC criteria.

Results: The median age of patients was 44 years of whom 32 had
squamous cell carcinoma. and 2 adenocarcinoma. Radiotherapy had been
used in primary management in 33, neo-adjuvant platinum chemotherapy
in 7 and previous palliative chemotherapy in 11. Isolated pelvic relapse
occurred in 21 (62%)., solitary extra-pelvic node relapse in 7 (20%) and
visceral metastases in 6 (18%). Median glomerular filtration  rate was
76ml/min.    Twelve patients received Ifosfamide  1.Sgmrnm2  and
Bleomycin 30 mg day 1-5, q 21 days. Twenty two patients received
Ifosfamide 5 gm/m2 as a single 6 hour infusion, q 21 days. Twenty-five
patients failed to complete 6 cycles of chemotherapy due to progressive
disease in 14, lack of symptom response in 2, and toxicity in 11.
Encephalopathy sufficient to abandon treatment occurred in 7 patients.
Thirty-two patients were evaluable for objective response. Pathological
complete response (CR) was achieved in 1 patient, partial response (PR)
was achieved in 3 patients. The objective response rate was 11.8%.
Symptomatic response throughout treatment occurred in eight patients
(24%), but of these objective response was seen in only 3 (1 CR, 2 PR)
and progressive disease in the remaining 5. Symptom response in these 5
patients may have been due to steroids. Response duration in the 4
objective responders was 25 months in the patient with CR and 4, 6 and
8 weeks in the 3 patients with PR All patients relapsed after reatment.

In conclusion: Ifosfamide, as given, is associated with unacceptable
toxicity and insufficient symptomatic efficacy, for use as a palliative
treatment in patients with relapsed carcinoma of the cervix.

P19         LUMBAR CEREBROSPINAL FLUID (CSF)

TO    SERUM      IHCG    RATIOS     IN   NON-
GERMINOMATOUS GERM CELL TUMOURS (NGCCT) OF
THE  PINEAL GLAND. 'P B Rogers, 1E Sims, '-2P N Plowman

'Dept of Radiotherapy, St. Bartlolomew's Hospital, and 2Dept of
Haematology and Oncology, The Hospital for Sick Children, Great
Ormond Street, London, UK

INTRO. In systemic choriocarcinoma, the ratio of spinal CSF to blood
DiHCG has been shown to be 1:286 (range 64-1546)1. Where the ratio
was less than 1:60, cerebral metastases were predicted. The pineal has
been clained to be disengaged from the blood brain barrier which some
authors believe accounts for the excellent response of pineal NGGCT
to chemotherapy If so, one would expect J3HCG secretion by the
tumour to have equal access to the blood.

PURPOSE: To find the ratio of unubar CSF to blood OIICG in pineal
NGGCT.

METHOD: Retrospective review of 15 patients with intra-cranial
germ cell tumours treated over the past 19 years.

RESULTS: In 4 marker positive pineal NGGCT boys (aged 14, 14, 17
and 19 years) the presentation lumbar CSF J3HCG levels were 140,
353, 17,500 and 1,390 IU/l respectively. Blood fiHCG levels were
respectively 21, 19, 1431 and 409 UA/ giving a mean CSF:blood ratio
of 10:1 (range 3-19. 1).

CONCLUSION: This figure is substantially lower than predicted from
the Bagshawe data on systemic choriocarcinoma and argues for some
privileged access of pineal NGGCT IHCG to the blood

REFERENCE:

1  Bagshawe KD    and Harland S. 1976    Immunodiagnosis and
monitoring of gonadotrophin-producing metastases in the central
nervous system Cancer, 38:112

P18         DOES LIPOSOMAL ENCAPSULATION OF

ANTHRACYCLINES IMPROVE RESPONSES
IN MESOTHELIOMA? A PHASE VII TRIAL.
JPC Steele. CA O'Doherty, M Evans, NH Gower, RM Rudd.

Dept. of Medical Oncology, St Bartholomew's Hosp, Lonxon
EC1, UK

The incidence of malignant mesotheltoma (MM) is projected to
increase 3-fold in the fwrst 2 decades of the Millennium. No
chemotherapy is considered standard but anthracyclines have
the best single-agent activity. Liposomally-encapsulated
daunorubicin (LD) has improved pharmacokinetics compared to
free drug. This study investigated the toxicity and anttumour
activity of LD in patients (pts) with pleural MM (120rmg/r2) in a
phase i/ll trial with dose escalation if MTD had not been reached
after 14 pts.

Patients with proven MM received LD (DaunoXome, NeXstar)
120mg/mn2 in 5% dextose IV q3 weeks (max 6 cycles). 13 pts
have been treated (M:F, 11:2; median age 54y, range 46-79; PS
0-2; histology: 8 epithe1oid, 5 mixed) of which 11 are evaluable.
Cycles of LD delivered were: 6 cycles (6 pts); 5 cycles (1 pt); < 4
cycles (4 pts: LD stopped due to disease progression). Grade
3/4 toxicities: neutropenia (1 1 pts), infection (3) and chest
tightness/flushing syndrome (2). Other toxicities: nausea (4),
fatigue (5) and anaemia (6).

Seven pts had disease stabilisation during LD therapy; 4
remain in SD a median of 4.7 moffiths from start of therapy
(range 4.2-8.2); 3 have progressed. There were no PRs or Crs.
In conclusion: (1) LD has not produced responses according to
standard criteria in MM; (2) the MTD of liposomal daurrubiAcin
is 120 mg/mn2 q3 weeks.

P20        LOW-DOSE STILBOESTROL AND H-DRO-

CORTISONE IN REFRACTOR) PROSTATE

CANCER, DC Farrugia, W Anseli, RTD Oliver, Medical Oncology,
St. Bartholomew's Hospital, London ECIA 7BE, UK.

Stilboestrol (SB) (3 mg day) is superior to flutamide as
primary therapy of metastatic prostate cancer (Chang et al. (1996)
JCO.14. 2250-7). and is active second-line treatment in up to 50?0 of
hormone refractorv patients (pts) (Dearnaley et al. (1997) BJC.76(1).
22). These studies reported thrombo-embolic events in 10-330/0 of pts
and up to 5% mortality from cardiac complications. In attempting to
reduce this toxicity. we added aspirn (75mg/day) to SB (1mg dap ) and
combined these with steroids (hydrocortisone 40mg dav). Xwhich are the
current standard treatment of hormone refractorv pts in the UK.

30 consecutilxe pts have been entered of which 2 u-ere non-exaluable. as
one died shortl- after entrv and the other mox ed to another centre. All
had PSA and or symptom progression on horrnone treatment. w-hich
Xwas discontinued prior to starting SB.

Miedian age >-as 69 (range: 51 to 85). SB wvas second line in 13. and
3 4' line in 15 pts. Median duration of treatment w-as 5.5 months
(range: 0.25-18). and median followx-up 7.3 months. With 21 pts still
aliv e and 15 still on treatment. Of 22 pts with data on symptom control
and PSA. 18 had symptomatic improv ement. 3 had stabilisation. and I
no benefit. Median duration of benefit PSA response xxas 5.6 months
(range: 0.5-12). PSA failed to respond in 2 pts. fell by 0-50%O in 4. 50-
900O in 9 and >90%    in 7.  The proportion with symptomatic
improvement in these four categories wxas 1 2. 2 4. 8 9. and 7 7
respectivel. Median time to PSA nadir u-as 4.2 months. and time to
progression (PSA or clinical) >-as 5.6 months. Toxicity included 1
mild cerebro-xvascular ev ent in an 83 y ear old. 1 upper-GI bleed. cardiac
failure in 2 pts. and 6 had fluid retention. Lo>- dose SB with hy dro-
cortisone has a fav ourable toxicity profile and is actix-e in refractorv
prostate cancer.  Randomised studies against hy-drocortisone are

needed.

34 Poster Presentatos

P21

AXILLARY IRRADIATION IS A POSTERIOR BOOST FELD

NECESSARY9 D. B.Landau', S. Kendall', RW. Laing', I.J. Laidlaw2
'St. Luke's Cancer Centre, Royal Surrey County Hospital, Guildford,
Surrey. GU2 5XX. 2 Frimley Park Hospital, Frimley, Surrey. GU16
5UJ.

Object To define the depth of the target volume when treating the

axillary lymph nodes in patients with early breast cancer and to confirm
the indication for a posterior boost field

Methods: 13 consecutive patients with early breast cancer who had

undergone breast surgery and axillary dissection by the same surgeon
were chosen. Radiopague clips had been placed along the dissected
axillary chain. At simulation of breast irradiation the patients were
screened in an AP direction and a wire placed (superior to inferior)

over the line of the clips. A centre of rotation was chosen along this

line at the mid-axillary point and AP and lateral were taken. The depth
of the clips from the surface wire was then calculated from these films.

Results: The mean depth of the most superficial clips was 51mm (range
37-69mm) and that of the deepest clips was 68mm (range 52-82mm).
Conclusion: A 6MV photon anterior field will not deliver adequate
dose to the axillary node chain. A postenor boost field is required

PFM          ADJUVANT THERAPY FOR RESECITABLE NSCLC.

JY. Douillard, FM. Delgado, JL. Lamrba, R. Rosell,
M. De Lena, J. Rodrigues, F. Carpagnano, P. Danel,
V. Kolek, F. Biville, IRPF Boulogne - France

Over the past 20 years, several studies have been carried out giving
chemoteray in an adjuvant sttig followng rsection of early NSCLC and
recently 14 clinical trials were analysed by  g  updated individual data
from 4357 patients(BMJ 1995).

This meta-analysis sugests that cisplatin-based regimens imrove survival
in rsected NSCLC, but the essential dnugs necesary for this beneficial
effect were not clearly identified. The results of randomised trials indicate
that the combination of Navelbie + CDDP is an active treatment for patients
with advanced NSCLC which may provide advantages compared with other
regimens and in many coumtries is considered as a reference regimen.

The ANITA project evaluates Navebibe (30 mg/m2/week over 16 weeks),
either as a single agent (ANITA 2) or in combination with CDDP (100
mg/n9 DI, 29, 57 & 85: ANITA 1) as adjuvant therapy for resectable
NSCLC, in terms of survival advantage in treated patients compared to no
treatment In addition, ANITA 2 will define the r6le of adjuvant
chemotherapy with a non-alkylating smgle agent and may extend the
expected benefits to patients not eligible for cisplatin-based regimens

More than 70 centres worldwide are particiating mi these trials mi which
1600 patients will be accnreL To date 512 patients have been included.

ANffA 1 (m= 402)    ANfTA 2 (= I116)

SEX M/F               89.7%/103%           85.7 %/14.3%

MediauAge (rag)       61(35/76)            62 (39 / 79)

StageI(S)/EIIA        35.8%/ 27.6%/36.6%   45.8%/19.6%/34.6%

60.9 % 39. %         61.7%/38.3%

pD           QUALITY ASSURANCE OF TARGET DEFINITION
P22          IN THE MRC RT-0I TRIAL OF HIGH DOSE

CONFORMAL RADIOTHERAPY (CRT) IN PROSTATE CANCER.

J Wilson. V Khoo, M Bidmead, E Aird. P Mayles, D Dearnaley on behalf of
The Quality Assurance Group of the MRC Radiotherapy Working Party.

Quality assurance is an essential component in multi-centre trials of CRT.

This exercise assesses clinician reproducibility in defining GTV, bladder, and
rectum in the MRC RT-01 trial.

Each participating centre received an instruction booklet with planning
examples. Clinicians were asked to outline GTV (prostate +/- seminal

vessicles/boost volume), bladder, and rectum on 4 non-contrast enhanced CT
planning cases. Stage, presenting PSA. and Gleason scores were supplied.

Outlines on CT films returned by 10 centres were transferred onto Cadplan by
a single clinician (JW). Each outlined structure will be compared for length
and position as determined by CT slice number. ICRU volume, and the
position of GTV isocentre.

The table below shows preliminary analysis of length (cm) and volume (cm3)
of target structures expressed as mean and (standard deviation):

Case 1       Case 2      Caw 3         Case 4

gt%=prost+base SV g s+SV  gti=prost+base SV  gt%=prost only
gtv length  5.1 (0-6)   6.2 (0.5)    6.8 (0.6)    5.3 (0.4)
gtv volume  47 (10)     83 (14)      79 (8)       56 (7)

bladder length 10.1 (0.4)  3.7 (0.5)  7.0 (0.2)  same as Case 3
bladdervolunx 958 (6)   118 (14)     253 (7)

rectal length  12.1 (0.7)  9.9 (1.4)  9.4 (22)   same as Case 3
rectal volume  74  (6)  138 (39)     137 (34)

These results show good consistency between clinicians from the 10 centres
with respect to outlining of GTV and bladder volumes. The greater

variability found in rectal length and volume has resulted in a clarification of
rectal definition in the RT-01 trial protocol.

Phase H pot stAdy of Cueyx (dozorubic  Ha,

FP24        pegy"AdIstwpegylMed' lipo smn) in paents with noperble head

a"     me&  squmnq  cdl canr. Harrington K",
Stewart S Haraison Da, Whitake Jb, Elliot Pb. Dept of Clii. Oncology'
Hammeisith Hospal, London, SEQUUS Phamaeus        Inc, Ul.

Purpose: The objectives of this study were: (1). to assess the
response of head and neck squamous cell cancer (HNSCC) to Caelyx
40 mg/n2 every 3 weeks. (2). to assess the toxicity and efficacy of split
course bd fractionated radical radiotherapy (RT) after 2 cycles of
Caelyx- (3). to evaluate the toxicity and efficacy of a synchronous third
cycle of Caelyx at alating doSes between 10 and 25 mg/M2 delivered
3 days before the start of RT.

Patients and Metbods: 11 patients (9 male, 2 female) with locally
advanced inoperable HNSCC suitable for radical RT received 2 cycles
of Caelyx 40 mg/rn2 every 3 week& Patients then proceeded to split
course bd fractionated radical RT (Phase I 38.4 Gy/24F/2.5 weeks,
two week gap, Phase II 25.6 Gy/16F/l.5 weeks). Response was
assessed clinically and by CT scanning in all cases. After 10 patients
had completed 2 cycles of Caelyx and radical RT, subsequent patients
also received a third cycle of Caelyx at escalating doses 3 days before
staring RT.

Resuls: After the first 11 aessable patients, the response rate to the
initial 2 cycles of Caelyx was 63.6% (7 PR, 4 SD). In 8 assessable
patients who have completed radical radiotherapy there have been 7
complete responses and one patient with a residual neck node. Two
patients have undergone neck dissection after completion of
radiotherapy - one pathological CR and one single involved node.
Caelyx has been very well tolerated; nausea and vomiting 0Y1 1, alopecia
0(1 1, oral mucositis 4/11 (median Grade 1), hand-foot syndrome 5/11
(median Grade 2), grade HI-Iv neutropenia (01 1. There have been no
tratment delays during radiotherapy and no increase in the incidence or
severity of acute radiation reactions. Updated information will be
presented.

Poster Presentations 35

Effect of tumour size on uptake of lllIn-DTPA-
P25         labeled stealth liposornes (IDLSL) in a tumour

xenograft model. Hamngton Ka. Rowlinson-Busza GA.
Abra Rb, Uster Pb, Stewart Sa. ICRF Oncolog) Unit. Hammersmith
Hospital, UKa. SEQUUS Pharmaceuticals IncO., USA.

Purpose: To examine the effect of tumour size on the uptake of IDLSL
in KB human head and neck squamous cell cancer xenograft tumours
(HNSCCXT).

Materials and Methods: KB tumour cells were grown in RPMI-
1640 medium containing 100 U/ml penicillin and 100 jgiml
streptomycin, supplemented with 10% foetal calf serum at 370C in a
humidified atmosphere of 5% CO2 in air. HNSCCXT were established
by injecting 5 x 106 cells subcutaneously in the flank of nude mice
which were used for experiment 4-20 days after inoculation, when
tumours of various sizes were present. Variation of liposome uptake
with tumour weight was studied in 62 tumour-bearing mice after iv
injection of 100 pl IDLSL (0.37-0.74 MBq). Mice were killed at 24 h
and IDLSL uptake of turnour and blood were measured by counting
weighed samples in a gamma counter. Also, the vascular volume (VV)
of HNSCCXT of various sizes was measured in 43 mice bearing 52
HNSCCXT (9 mice had bilateral tumours implanted 10 days apart).
Quadriceps muscle was used as the control tissue.

Results: Mean % ID/g of IDLSL at 24 h was 7.2?6.6 for tumour and
0.3?0.1 for muscle. There was an inverse correlation between tumour
weight and liposome uptake (rs = -0.62, p < 0.001). Uptake for
HNSCCXT < 0.1 g, 0.1 - 1.0 g and > 1.0 g were 15.1?10.8, 5.9?2.2
and 3.0?1.3 % ID/g, respectively. There was an inverse correlation
between tumour weight and tumour VV (rs = -0.60, p < 0.001) but no
correlation between muscle weight and muscle VV (rs = - 0.02, p >
0.1). The paired tumour samples showed significantly greater VV in the
smaller of the two tumours (p = 0.02). For 20 paired muscle samples of
different weights, there was no such correlation (p > 0.05).

Conclusion: Well-vascularised tumour deposits are likely to be more
effectively targeted by Stealth liposomes than larger, poorly-
vascularised tumours. This has important clinical implications in
developing liposomally-targeted therapies.

Stealth liposome encapsulated doxorubicin (SLEDW

P27        and cisplatin (SLEC) for targeted radiosensitisation

in xenograft turmours. Harrington Ka, Rowlinson-
Busza Ga. Uster PI. Stewart Sa. ICRF Oncology Unit, Hammersmith
Hospital, UKa. SEQUUS Pharmaceuticals Inc, USAb.

Introduction: The clinical development of radiosensitisers has been
impeded by: (1). dose limiting systemic toxicities and (2). sensitization
of normal tissues in the radiotherapy field. Stealth liposomes effectively
target head and neck cancers and alter the pharmacokinetics and
biodistribution of encapsulated agents offeming selective tumour
delivery of radiosensitisers, enhanced therapeutic effect and reduced
systemic and local toxicity.

Purpose: To evaluate the efficacy of SLED and SLEC, in comparison
to unencapsulated doxorubicin (UD) and cisplatin (UC) as radiation
sensitisers in human head and neck squamous cell cancer xenograft
tumours (HNSCCXT).

Materials and Methods: HNSCCXT were established in nude mice
by subcutaneous injection of 5 x 106 KB cells. Tumour volume was
calculated as r/6.dl.d2.d3 from three orthogonal diameters thrice
weekly. Tumour volume on the first day of radiotherapy (RT) was
defined as VO and was subsequently expressed relative to VO. Time
taken to reach 3VO defined a surrogate measure of survival. Groups of
10-12 mice received iv injections of 50 jg of unencapsulated (UD or
UC) or liposome-encapsulated doxorubicin or cisplatin (SLED or
SLEC) 16 hrs before single fraction RT (4.5 or 9 Gy).

Result: Median   es to 3VO in  s were as follows:

No drug67127                              2.
UD 50 Xg          9.           652
SLED So   g        157         246     a     5

UC So PR          9.4         12.9        18.6
SLEC 50            10.2        21.2        33.9

Conclusions: Low-dose SLED and SLEC enhance the effect of
single fraction RT in HNSCCXT. Each liposome-encapsulated agent
was significantly more effective than its unencapsulated counterpart .

26       Stealth liposome entrapped doxorubicin (SLED) and
P2         cisplatin (SLEC) versus head and neck xenograft

tunours Harrington Ka, Rowlinson-Busza Go. Uster PA.
Stewart So. ICRF Oncology Unit, Hammersmith Hospital. UKa. SEQLUS
Pharmaceuticals Inc., USAb.

Purpose: To study the effect of SLED and SLEC. compared to
unentrapped doxorubicin (UD) and cisplatin (UC), in head and neck
squamous cell cancer xenograft tumours (HNSCCXT).

Materials and Methods: Groups of 10-12 nude mice with
HNSCCXT received single iv injections of one of the following agents:
SLED, SLEC, UD or UC. Control animals received no therapy. The
tumour was measured in 3 orthogonal diameters (dl, d2, d3) and the
tumour volume was calculated as V = 7r6 dl.d2.d3. Tumour volume
was assessed on the day of treatment (VO) and then 2-3 times per week.
Mice were killed when the tumour had tripled its original volume (3VO).
Time take to reach 3VO was used as a surrogate measure of survival.

Results: The median time taken in days for tumours to reach 3VO are
displayed below. The median time to 3VO for untreated controls was
7.3 days.

AGENT     50   g    10 0      200 X     250 R   | 500 Ilg
SLED       15.4      21.9     40.6       NID       ND
UD        9.7       8.7      10.4       ND        ND
SLEC       10.2      15.9      ND        24.6      32.5
UC        9.4       8.5       N         15.3      NK*

Key: ND = not done, NK* = not known (all mice died five days after
treatment).

Durable complete response or stable disease (>60 days) was seen after
200 gg ST E  in half the mice. No toxicity was seen with single dose
SLED or SLEC.

Conclusion: SLED and SLEC show significant activity in
HNSCCXT. Both SLED and SLEC were more active than their
unentrapped counterparts. Clinical trials of both agents in patients with
head and neck cancers are planned.

Stealth liposome encapsulated iododeoxvuridine

P28        (SLIUdR) for targeted radiosensitisation in xenograft

tumours. Harrington Ka. Rowlinson-Busza Ga. Uster Pt.
Stewart Sa. ICRF Oncology Unit, Hammersmith Hospital. UKa. SEQUUS
Pharmaceuticals Inc. USAb.

Purpose: To evaluate the efficacy of a novel Stealth liposome-
encapsulated acylated derivative of iododeoxyunrdine (SLIUdR) as a
radiosensitiser in human head and neck squamous cell cancer xenograft
tumours (HNSCCXT).

Materials and Methods: HNSCCXT were established in nude mice
by subcutaneous injection of 5 x 106 KB cells. Tumour volume was
calculated as 7rJ6.dl.d2.d3 from three orthogonal diameters thrice
weekly. Tumour volume on the first day of radiotherapy (RT) was
defined as VO and was subsequently expressed relative to VO. Time
taken to reach 3VO defined a surrogate measure of survival. Groups of
10-12 mice were treated according to the following protocols: (1). 600
fig of either SLIUdR or free LUdR (flUdR) was given iv 16 hrs before
single fraction RT (4.5 or 9 Gy). (2). either SLIUdR bolus loading
doses of 4 x 300 jg were given on alternate days for 8 days or ftTdR
1200 jg was delivered by 8 day subcutaneous Alzet pump infusion
before fractionated RT with 9 Gyi3 fractions/3 days.

Results: Median times to 3VO in days were as follows:

No xR     4.5 Gra v/ F   9 Em/F  9 Gy/3F/3d
No drug        .7      127        226    _    3
flUdR "Og        8.2      17.6      31.5       N

_L~R   O      8.9     12.4       22.7       N

fldR10U         7.9      NND                  17-6
SLIUdR 4x          8.8      ND        ND         21.6

Conclusions: SLIUdR given according to a loading schedule
enhances the effect of fractionated RT and is more effective than fTUdR
in HNSCCXT. The effect is not evident after single doses of SLIUdR
before single fraction RT.

36   PcstePresentaUons

Biodistribution and pharmacokinetics of lllIn-

P29          DTPA-labelled stealth liposomes (IDLSL) after intra-

peritoneal injection in mice. Harrington Ka, Uster pb,
Stewart Sa. ICRF Oncology Unit, Hammersmith Hospital, UKa. SEQUUS
Pharmaceuticals Inc., USAb.

Purpose: To examine the biodistribution and pharmacokinetics of
IDLSL after intraperitoneal (ip) injection in mice as a means of studying
their potential application as a vehicle for intrapentoneal delivery of
cytotoxic drugs.

Materials and Methods: Nude mice received an ip injection of 100
p1l of either IDLSL or 11In-DTPA, each containing 0.37-0.74 MBq of
radioactivity. Mice were kiRled at various time-points and a wide range
of tissues were dissected and their content of radioactivity determined
by counting weighed samples in a gamma counter. Standards of the
injectate were counted to correct for physical decay of the radioisotope.

Results: Data are shown below in % injected dose per gram (% ID/g).
Table 1. Biodistibution of IDLSL

Tissue    1 h      4     h  18 h      24 k     48 h )        h

Blood   8-2 ?5.0  22.7 ?3.4 10.1 ?3.6  6.3 ?1.0  1.7 ?0.6  0.03 ? 0.0

Perit  10.3 ?8.81 6.5 ?5.6  5.4 _2.3  3.4 ?3.9  2.0 ?0.7  1.3 ?0.51
Liver   2.6 ?1.0  8.6 ?0.9  16.8 ?3 6  16.1 ?3.5  153 ?4.8  10.7 ?2.6 J
Spleen  3.4 ?1.4  8.9 ?1.11 12.3 ?1.6  11.7 ?1.9 13.1 ?2.7  10.7 ?_13
Kidney  2.9 ?0.9  5.3 ?1.0  5.8 ?1.6  45 ?0.6  5.1 ?1.13.8 ?0.5
Lung    2.2 ?0.9  6.2 ?1.6  2.9 ?0.7  2.0?05 |1.00.1   05 ?0.1

Table 2. Biodistribution of 11 lIn-DTPA

Tissue   5 min   30mm       1 h       4        24 k       72 k

Blood   3.3 ?1.2  0.9 ?03  0.2 ?0.1 0.02 ?0.00 0.00 ?0.00 0.00 ?0.00
Perlt  12.8 ?4.1  0.9 ?0.2  0.5 ?0.2 0.04 ?0i.O  0.03 iO.01 0.03 ?0.02
Liver   3.4 ?0.9  0.4 ?0.2  0.2 ?0.0  0.2 ?0.2  0.05 iO.0 0.06 iO.04
Spleen  6.5 ?2_7  0.5 ?03  0.2 ?0.0  0.05 ?0.01 0.05 ?0.01 0.00 ?0.00
Kidney  7.1 ?1.4  2.6 ?0.9  1.7 ?0.1  0.7 ?0.1  05 ?0.1  0.3 ?0.0
LuuZ    2.5 ?0.6  0.7 ?03  0.2 ?0 1 0.03 ?0.01 0.02 ?0.01 0.00 ?0.00

Conclusion: Encapsulation within a Stealth liposome matrix
signifcantly alters the biodistribution and pharmacokinetics of 11 In-
DTPA. IDLSL are rtained within the peritoneal cavity for a prolonged
period. Stealth liposome-encapsulated drugs offer a novel treatment
approach for diseases confined to serous cavities (eg ovaran cancer).

P31          CUrA        SCOPY)

FED      IN THE NunuTNA MANAGEMENW OF PATUNS
WTIH ORAL CANCERS TREATED BY RADIOTHERAPY.

N.Gilbe1t, HYPeky', S.Bullrd2, S WhiakW . 'Dept of N trim & Ditdcics 2Dep of
ObIgy, Tbe Royal Sey Couxnty Hotal, Guilfd, SUTy GU2 sXX

Head an neck cance patients are often malnoished at d     i,  h
routine tatmtte                 obl     (Cl    k  and Mossman
1983). Gilbert and Petey 1995, de        thed       a   role of
PEG feding in rcoveig weight loss post-operatively and

adequaten                 radiotherapy. Early PEG feeding rema

rarely prescribed. Five ubjects, mean age 60yrs (45-75yrs) with oral
squamous cell           to undergo radical radiotherapy were each
fitted with a PEG tube prior to treatment.

A  Pvuced at DbWoo
1 Nu     au

(a)  -_

Grp   iUC     i5Fay

(b) N-7tmcl m - & y 7 dy food wv
(c) B-ioy- FBC/am

2. QNWy of We pn

3. T,w m

Al abjects exeienced treatment complications limiting oral intake.
Four subjects     ained or improved their nutitional intake with

sunkmentav PEG fpeinc,

*U TE ITI  * T U AL  T A

g 1  "a   "

:::j      J   J    L It.., I

Sulementary PEG feeding can prnmote optimal tient intake when
requirements are high and oral intake limited. Further tials are required
to    blsh clnical benefit firom early intervention.

1 D  J--   _ K-L (1983) Cr 51, 811415

GAt NS A PqkY H.F. (1996) Prc No Soc. vl 4 ?No 3 1UA

'Cwsry Hid oDqA of Dc a NaWSWO. NOaM&=COy Hoqitatl  _   NGS PB.

P30

A NURSE LED PERIPHERALLY INSERTED CENTRAL UNE

CATHETER (P.I.C.C. UNE ) SERVICE - A REVIEW OF THE FIRST
YEAR

S. Moat ', P. Murray ',T. Hickey 2, M Boots 2,B. Sizer '. S. Tahir '.
I Department of Clinical Oncology, Essex County Hospital,
Colchester.

2Department of Haematology, Colchester General Hospital,
Colchester.

P.l.C.C. lines are increasingly used to facilitate the administration of
chemotherapy, serial blood sampling and blood product / antibiotic
support in cancer patients.

From December 1996 we have established a nurse led P.l.C C. line
insertion and maintenance service. In the first twelve months 60

P.l.C.C. lines (45 single, 15 double lumen ) have been placed in 50
patients. The patient age range was 49 years ( range 31 - 78 ). In 5

additional patients cannulation was unsuccessful. 54 Of 60 lines were
piaced with a singe venupuncture. The median duration of P..CC.
line pacement was 59 days with a maximum of 344 days.

The incidence of complications was clearly related to experience. In
the first six montis premature removal of lines occurred in 10 of 20

lines (50%): 5 infecbons, 2 thromboses, 2 migration, 1 leakage. In the
second six months only 3 of 40 lines required removal due to
complications (0.07%): 1 infection, 1 thrombosis, 1 migration.
P.I.C.C. lines are a safe and reliable method of establishing

continuous venous access for oncology patents and can be routinely
placed and maintained by experienced cytotoxic nurse specialists

P32

am OSEWrlyLTY Of    INE     OC&DCIK   CELL Lim  IS
a IImACl or PISBYCI     WIl Sr=5.

L.S1., I.J.Iasa, NE.C.Biby & J.Doule. Cliica

Iogy blit, bkitsity of kadford, lest Yorksire.
u6LA. D7 lDP.

Cel lines with defects m MMR have been linked with in vitro
toleance to DNA        agas includng cs-platin and 6-

ine (6-TG). In  dmanrSiste to ese annt-tumour
compnds h        also b   m   ed wih MMR defects. Vmble
M       defecdiv models are neede to undetad any possible
relationslp be    M MR dysuction and ce in
dinical con nditionas sicha as hereditary non-polyposis cokorcta
cancr (NPCC).

Compaison wee made      re between IC  vals aftesr

expoawe to cs-platin and 6-TG on mouse adxcacnm     cell
lins MAC 13, MAC 15A and MAC 16          IC" values for

HCT1 16 and DID- 1, hmun colon           w   swith known
MMR    Imitti ns at u LJ and G1BP    eve. The MT

ay was usd after 72 har coetimou exposue and p      a  ge
cel aviva taken as the rato betwen the obanc of treated
cedls and the untreate control cells x 100.

Mean IC" vale (n-=3) for the HCT1 16 cel line showed a

uy gr nae (p < 0.05) to both                spain    and 6-
TG cared to al of the MAC cel lines Ranges varied from a 4
fold diffeRCAs ins-platin c masitvi with MAC 16 to an
18 fol Minmce with MAC 13. MAC 13 cdl line was alo very
seiti   to 6-TG,       HCT1 16 over 40 t   moes ne reisunt.
HCT1 16 was              5 t     more       to 6-TG thn
both MAC 15A and MAC 16. Simdi             were seen
betwee DID-I and the MAC cl ine.

None of the MAC ce lines dil     iilar c       stane
to is-platin or 6-TG cmpaed to the lman cel lines,_
they may be MMR cmpetent.

Poster Presentatons 37

p33          PHASE I STUDY TO EVALUATE THE

TOLERABIUTY OF CAELYX (DOXIL) IN
COMBINATION WITH PACUTAXEL IN

PATIENTS WITH METASTATIC CANCER. A.L. Thomas'. R.

Langley', J. Carmichael', P.Woll1, E. Mason1, H. Welbank2.

CRC Dept. of Clinical Oncology, City Hospital, Hucknall Road,
Nottingham', SEQUUS Pharmaceuticals Inc., London2.

The combination of doxorubicin and paclitaxel in the
treatment of metastatic breast cancer has produced
encouraging results however, early reports have demonstrated
cardiac toxicity in some patients. CAELYX is a formulation of
doxorubicin where the drug is encapsulated within a pegylated
(STEALTH) liposome. This formulation may overcome the
limitations of conventional doxorubicin when used in
combination with paclitaxel.  16 patients with confirmed
metastatic cancer were treated   with a combination   of
paditaxel (175mg/m2) as a 3 hour infusion every 3 weeks and
CAELYX every 6 weeks for up to 6 cycles. The initial dose of
CAELYX    60mg/iM2  resulted  in  dose-limiting  mucositis,
neutropenia and planter-palmer syndrome, therefore after the
first 5 patients the dose was reduced to 50mg/m2. In 14
evaluable patients there were 6 partial responses (5 breast, 1
adenocarcinoma), 4 stable disease, and 4 progressive disease
(2 patients were stable at 12 weeks but subsequently
progressed). G-CSF was only administered on one occasion,
haemopoeitic support was not routinely required.    Left
ventricular ejection fractions were measured at baseline and
end of treatment.  Only 1 out of 7 patients recorded a
significant decrease in left ventricular function but this
remained within normal limits. The trial is ongoing with an
additional 10 patients being treated with CAELYX 30mg/m2

every 3 weeks in combination with paclitaxel 1 75mg/M2.

p35             MIRI IN RADIOTHERAPY PLANNING OF

THE PELVIS. S.A. Dunne. A.S. Gee.
MRI Scanner, Bristol Oncology Centre, Bnrstol BS2 8ED

PURPOSE To incorporate MRI scans of the pelvis into the
radiotherapy planning system. To identify useful and relevant
sequences to enable more accurate tumour delineation.

MATERIALS/1METHODS 20 patients undergoing routine
radiotherapy treatment planning for tumours to the prostate or
bladder were selected. Informed consent was obtained. In addition
to the standard CT scan used for planning their treatment, these
patients under went an MRI scan of the same region.

Patients were scanned in the radiotherapy treatment position on a
special table insert with MR markers placed on their tattoos as in
CT.   A variety of sequences in different orientations were
performed for review by the Oncologists.

RESULTS A suitable sequence with minimum distortion that could
be corrected for geometric distortion was identified.  After
correction these images were transferred to the radiotherapy
planning computer. These were then viewed alongside the CT
scans by the oncologist to identify the target volume to be treated.
Other sequences useful for delineating the target volume were also
identified.

CONCLUSION Planning scans can be corrected for geometric
distortion and successfully transferred to the radiotherapy planning
computer for viewing alongside CT images. MRI can be used in
place of radio-opaque contrast and fluoroscopy to enable the apex
of the prostate to be accurately identified. A sagittal midline image
was found useful for bladder tumours and coronal images were
identified as being most useful for prostate localisation.

INTRACELLULAR ANTIBODIES

P34          (INTRABODIES) DIRECTED AGAINST THE

KINASE INSERT DOMAIN OF KDR
M. Stoddakt P. Hewt L Durrut and J. C. MFmy.

CRC De     pmt o dkical oncology, City hospitL No , NG5 IPB

Vascular tar      Of tumolrs relies upon expression Of tumour-
endotheRial specific tas. It has been s       t   h    the

cpor tyr       i dnas      KDR ad Flti fulfil fts crftrion.
Before the   I ftd pienial can be e ited, the precise role and
mechanism of action must be elucidated. Wth this aim in mind
we are producing mxnodknal alibodies a          the h   y
conserved kiase insert domaIs (KID) of KDR and Flt 1. Initially
tese afibods    can be used to loate te receptors in tissue
secions. Later the aim is to clone the FragmeIre antg   bindig
(Fab) re       and express hem   intracelk  y (inacellula
antbodi     or intraodiies). These wUld serve to block fe
function of an inividul       r  and alow any ph     pic

hangss to be osedrved. A unique 16AA sequene hfo   te KID
Of KDR ws stecaIy p         d   and used as an immunogen.
Hybtidomas were prod        and the fconitoned media wes
EUSA-sceened agast the immunsing peptde and whole
cels. Of th  28 posive weNs a representativ          were
coned and te remind       frozen down. D11.0.6 is an lg2b,
which has been shown to read strongly a  nst the pepbde. It
also reacs wift HUVEC cels to a titre of 1:512 (furhe diluAons
have not been attd). The actvity of Dl1.0.6 shows a
patter of non-spci bi      a    s COS cels which fitres out
aftr 1:32. Thus at a dilton of 1:100, DI 1.0.6 reacts s fically
agans HUVEC in an EUSA screen assay. Steps are beiN
taken to optimise an immnfic19-_ie regfme and screen

Jsqt Oda    cell types. P   _ei*y    data s1ows HWNEC
staining  with the a  dy   a    t   KDR, is punctate and
uniormlv distributed over the whole call.

P36          PHASE  II ENALUATION    OF  HIGH   DOSE

ACCELERATED RADIOTHERAP FOR LOCALLY

ADVANCED    AND   ANAPLkSTIC   THYROID   CARCINONMA. G.
MNitchell, R. Huddart and C. L. Harmer, ThN-roid Unit, Rox-al Marsden
NHS Trust, Fulham Road, London SWV'3 6JJ, UK.

AIM:   Anaplastic thyroid cancer (ATC) responds poorly to
conventional radiotherapy and prognosis is dismal. The majorntv of
patients die with uncontrolled local disease. We in-estigated the use
of accelerated radiotherapy aiming to improve local control in
patients with ATC. Toxicity was assessed prospectively- for all
patients with thyroid cancer treated with accelerated radiotherapy

METHODS: 25 patients were treated, of whom 17 had ATC and
the remainder other advanced thyroid carcinomas. Patients wvere
treated twice-daily, five days a week to a total of 60 8Gv in 32
fractions over 20-24 days.

RESULTS: Patients with ATC demonstrated a significant response
rate of 58?o. 2/17 (11%o) a complete clinical response, 8/7 (47%.) a
partial response and the remaining 7 (41%o) had stable disease.
Median survival of 10 wveeks (range 7 days to 31 weeks). Local
control was maintained in 15/17 patients (88?o) until death from
progressive metastatic disease or concurrent illness Only 2 /17
patients died with progressive pniman- disease. In all 25 patients
toxicity was high with 18 patients requiing a fluid diet or IV fluids,
NG tube feeding or opiate analgesia.

CONCLUSION: This approach improved the response rate to
radiotherapy but toxicity was high. A modified accelerated
radiotherapy protocol is currentlv being explored Improved local
control did not improve sur.-ival from anaplastic thyroid carcinoma
M these patients.

38 Poster Presentatons

P37

PALLIATIVE RADIOTHERAPY IN NON SMALL CELL LUNG CANCER - TO
RETREAT OR NOT TO RETREAT A' XRREA :.". -SrOER AND      50L.SEP
WEFGON PAU -OSPr7A, W41tAe ROAD., 51E  ?  25.

:;A  V   RA )O7-:'R~' A..2VATES S       N A .A95GE 7ROPOwrON OF
PATIENTS WTH ADVANCED NION SMki. CELU LUJN(5G ANER (N5CLG)_ HOW'EVER
SYMPTOMS MAY SUBSEQUENT.y ;ECUR AND THER 15 NC O..i CONSU5 ON THE
ROE OF 9UErTHER RADOT-ERAPV'. N PATCJLAR THERE 5 SOME CONCGERN A5t7
NOREASED TOX C7y, E5PECAY THE  5 OF RAD2A'CN 'YE.7S

1TH05 BESTWEEN AU&U5T 1994 AND 0OCBER 15 30 PA7lENTS UNDERWENT A
5ECOND  COURSE OF PA.LATVE    ADCOTHERSY  FOR N&2.C. THEY WERE

ZOSPCVEl Y    2./ED   T7O EVALTE THE EFC.,' AND TOXC57      OF
RET EAT9EN` 'NAL ASSE55'ENT5 WERE PERORLED PRZOR 'C RADCOTHERA

WAENS WERE REASES5ED     ONE "ONT7' AFTER 'REATNT ANDC6 MNT-LY
HEREFTER THE MED~A A6E WAS 70 -2EA.5 (RANGE 45 - B9 YEARS) AND THERE WAS-
A    E   "ALZE WEPONDERANCE (2r. 3'> THE NTE7VAL SEWEEN PRZ5 AND
5ECOD COUR5ES OF RACTHERAPY RANG5EL   5-O42 -I' - "ONT-5  N

'RES2E5D DOSE FOR RE'REA"'ENT WAS 3  %S N 'S DAy -7A(T5 ON 09C%6'-' O-
PATIEN7 W BEEN TREATED WT!H 17GY IN 2 WEEY RACTONM A-A'v.

E5S,,5 A 6'15- PROPOZ'ON OF PATE-NT5 REPORTED MPRNM'1EN  N 5V .51"
-OEP'55 (63%) AND 13  PA% 54N2% RESPONDED.1 '057 FREUE' 5  $

;ESPONSE RATES WERE A-5C SEN FOR 7D'SPNOEA '60%> D 7"S2 A (5 A%( AND

UGH (52%t. TREATENT WA5 W:-', OL3ERATD W, AND M05T REPORTE3 5DE
E=CT5 WEE MiNOR. THERE WAS ONE CASE OF SYMPTOA7C      RAZ7ON
PNEjMON1LS AND ONE OES5OF2AE  S7rO"ZRE S  .5?1s COR DAMAGE WAS NC'
5EEN.

CLU5 ON THIS S5L STUDY HAA5 50WN THAT A 5ECON CURSE OF PAILATVE
RAZOTHRAPY CAN BE WELL OLERATD AND PROVXDE USEUL RELEF OF SYMPO5
FJURTHE RESEAO  S5 NEEDED 'O DEPNE THE R-OE OF 'J  '.E JRSES 5-
:A..AV RA:_     _R" N NSC..O

DS~n     DOSE ESCALATION USING CONFORMAL TECHNIQUES FOR
P39         OESOPHAGEAL CANCER IN THE SETTING OF

CHEMOTHERAPY-RADIATION. Viviers L', Bedford j2, Guzel Z.
Tait, D', Childs p J 2, Webb S2, Oldham M2, Nahum A E2

'Academic Department of Radiotherapy ard 2)oint Department of
Physics, Insttute of Cancer Research and Royal Marsden NHS Trust, Downs Road,
Sutton, Surrey SM2 5PT, UK.

Backaround

Local failure and distant metastases both contribute to poor
treatment results in oesophageal cancer and provide the rationale
for attempting radiotherapy dose escalation in the setting of
chemotherapy-radiation.

Method

CT planning data for five patients with oesophageal cancer was
used to compare three treatment techniques, two-phase
conventional (CV-2), single phase conformal (CF-1) and 2-phase
conformal (CF-2), in terms of normal tissue (mean lung) dose.
Constraints imposed were to limit cord dose to 45Gy and 48Gy,
and lung outside AP-PA beams to 18Gy. For each technique PTV
Doses of 55, 60, 64 and 7OGy were attempted.

Results

Mean Luna Doses for 45Gy to SDinal Cord.

TARGET Dose (Gy)  Mean lung dose - Mean lung dose - Mean lung dose -

CV -2 (Gy)      CF-1 (Gy)       CF -2 (Gy)

55             14.8            16.1            11.0
60             17.4            17.6            13.3
64             19.4            18.8            15.1
70             21.0            20.5            16.1

CF-2 was superior at each target dose level when limiting the cord
dose to 45Gy or 48Gy.

Conclusion

CF-2 technique would allow dose escalation of 15% compared
with CV-2, keeping mean lung dose at the same level, and
delivering a target dose of 7OGy (27% dose escalation) would

involve only a 9% escalation of mean lung dose.

P38         10.8 MG GOSERELIN EVERY 13 WEEKS FOR

ADVANCED PROSTATE CANCER. MF Sarosdy', PF
Schellhammer, MS Soloway, NJ Vogelzang, ED Crawford, J Presti, P Kelley, P
Mitchell, L Porter. *University of Texas Health Science Center, San Antonio

Objective: To determine the endocrine effects, efficacy and tolerability
of a 10.8 mg depot formulation of goserelin acetate (ZOLADEXTm), a
luteinizing hormone-releasing hormone agonist analogue, administered
every 13 weeks to patients (pts) with advanced prostate cancer.

Methods: Between July '95 and May '96, 59 pts with locally advanced
(T3, T4) or metastatic prostate cancer enrolled in this open-label,

multicentre trial. Testosterone measurements, PSA response (?90% of
the baseline value or a decrease to <4 ng/ml), subjective response (pain,
analgesic use, performance status), and objective response (National

Prostatic Cancer Project criteria) were 10 efficacy endpoints. Quality of
Life (QOL), a 20 endpoint, was assessed by a questionnaire.

Results: Mean testosterone conc. decreased to <30 ng/dl by week (wk)
4, remaining so for the treatment duration. No statistically significant
difference in mean testosterone conc. was observed between wks 12
and 13 or wks 25 and 26. There was adequate suppression of serum

testosterone in 57/58 (98%) pts at wk 13 and 51/52 (98%) pts at wk 26.
Of the evaluable pts, 52/58 (90%) had a PSA response; 6/11 (54%) had
a subjective response; 45/58 (78%) had a partial objective response, 3
(5%) had stable disease, and 10 (17%) had objective progression.

Except for a significant (P=0.014) decrease in overall sexual interest,
QOL remained unchanged during therapy. The most common

treatment-related side effects were hot flushes (60%), sweating (12%),
decreased libido (5%), and impotence (5%). Mild injection site
complaints occurred with only 3/221 (1%) depot injections.

Conclusions: The 10.8 mg depot formulation of goserelin acetate

provides adequate suppression of serum testosterone, produces PSA
responses, subjective responses, and objective responses, and is well
tolerated when administered every 13 weeks to pts with advanced

prostate cancer. (ZOLADEX is a trademark, the property of Zeneca
Ltd.)

P40

A STUDY TESTING THE ROUTINE USE OF ULTRASOUND

MEASUREMENTS WHEN SELECTING THE ElECTRON ENERGY
FOR BREAST BOOST RADIOTHERAPY.

S. J. Heew ', E Moskovic 2, L  aS&,  2 end J. R. Yamrdd'
1w & R              2dWpt of Diagnostic Rao   , Ro

Mwisdon HosaI_ MHS Trust and Irsikute of Cancer Re,
Suon SW2 FT

The rot*ie use of high freqtency ultrasound was estabshed for
meaimng te dept of te bwnr bed when plar*g brat boost
raialerpy. 52 dely       post operatw breas caKr pabers
had boh the    nenmd cbini aeMent of Ons depth eW en

weas    d assessment pedolmed. The    iasod do -       used
as tn gold stEndo   . For eact assessed dep  an elemon  ergy
was dlosen from    e keblevw  de;hldonse    . 54% of pants
needed1 toeir eledron energy diengd from, VWa dhose by Acincl
assessment. The tble shg  s the numer of paiet   (categorised
by bra size) aganst the changes mad  to the*icyasse
eiecbw energw (E).

Bra size  No.paiens E. hIcreased  E Decreased   E. Sm
Al        52         14 (%)       14(27%)       24(48%)
Lage      13          4 (31%)      1 (8%)        8 (61%)
Med=n     31         10(32%)      11 (38%)       10(32%)
Smal       8          0            2 (25%)       6 (7%)
Certin brne emrraged when breast size wqls considere& Pads

with large bras  more ofn redn ed    n enrgy to be increaed
whereas te patie-ts wih     md   breato s  -add   an energy
lrecion.   ft was cnckaded out   edF use of    as   d,     n
ibied    m   pFenningVe     raiohe  y   breat boos    is a

b, safe end w    ai mehd Of        Xte a         Ofy o
m    kre   a    .

Poster Presentations 39

HEPATIC NECROSIS FOLLOWING

P41            INTRAHEPATIC ARTERIAL 5-FLUOROURACIL

AND FOLINIC ACID. C.L. Hanna. M. Bourne.
M.C.A. Puntis and T.S. Maughan. Velindre Hospital NHS Trust.
Whitchurch, Cardiff. CF4 7XB.

In metastatic colorectal cancer with isolated liver metastases. regional
chemotherapy using the intrahepatic artenal route is of interest because of
the hope that high doses to the tumour may be achieved without
corresponding increases in systemic toxicity. Since 1995. twelve
patients in our centre have received this treatment. All patients had
histologically proven adenocarcinoma with disease confined to the liver.
Patient characteristics included mnedian age 60. range 35-75: performnance
status 0 or 1; primary site colon 8, rectum 4; sex male 9, female 3. Two
patients received previous 5-fluorouracil based chemotherapy.

Treatment was intravenous folinic acid 200mg/M2 over 2 hours
followed by intrahepatic arterial 5-fluorouracil 400mg/m2 over 15
minutes then 1 .6gm/m2 infusion over 22 hours. This was repeated over
the next 24 hours. The schedule was given every 2 weeks. Patients
received 1 to 13 cycles of chemotherapy, median 6. Four patients
received fewer than 6 cycles (1 portacath malpositioned. I inadequate
perfusion of liver. I extravasation and 1 toxicity).

Eleven patients were evaluable for response. The overall response rate
was 36% (2CR. 2PR). Three patients (27%) had SD. Four patients
(36%) were treatment failures (3PD and I toxicity (episodes of confusion
when using portacath)). Four other patients had grade 2 or more toxicity.
The unevaluable patient had unmeasurable disease. The mfedian duration
of response was 14 months, range 6 to 18 months. Ten patients (83%)
are alive after mfedian follow-up 12 months, range 5 to 32 months.

Three patients (25%) developed extraordinary large volume cystic
changes in the liver in areas previously free from metastases. These
became evident after4, 6 or 12 cycles. In one patient green turbid fluid
was aspirated under CT guidance. In another. biopsy showed necrotic
tissue. These changes were consistent with hepatic necrosis secondary
to chemotherapy. On subsequent serial scanning these lesions gradually
diminished in size and had no effect on liver function or performance
status.

Intrahepatic arterial chemotherapy appears to confer a higher response
rate in metastatic colorectal cancer than intravenous regimens.
Randomisation into the current MRC trial CRO5 comparing innahepatic
arterial and intravenous chemotherapy is recommended.

CERV&NAFEERVING TFEATMENT OF ADVANCED ORAL AND
CROPHA.RYNGEAL  SQUMOUS      CAFrINOAMAS  USING
P43                CHEMOT1}EAPY AND RADIOTEAPY - A PLOT STUDY. C. D

Saase1 R P. Cretn2, P. J. Baak, S. E. Fi  1. H. McVcx3. 0 A
L   .    Dept of CWual Oncokgy, Cly HolA, No q$  2Do d Cical OrcooW. PooleGenerG
I Dorset H3     & Ne& Ckw. Qeen's MecK2 Cerie, No,N

Obiee

Randonised trials have shown that cisplatin/lluorouraci chemotherapy followed by
radcal radiodwrapy for responding paterts is an approach that gives local control
results as good as those achieved by curative surgery for squamous carcinoas of the
larynx and hypopharynx. while preserving organ function. Localy advanced oral cavity
and oropharyngeal squamous cei          present a considerable therapeutic
chalenge. Surgery, whilst potentially curative, resutts in signifcant functional morbidiy.
We thus sougtt to evaluate the possble role for neoackuvant chemotherapy (CT)
folowed by radical radiotherapy (RT) in patients with such tumours.

NWhod

So far, 11 patients with localy-advanced (inoperble, or operable only at the cost of
severe functial disabdity) squarmous carcinoma of the oral cavity (5) or oropharynx
(6) have been treated- One patient decined further chemotherapy afterthe frst cycle,
al others received a minrrium of 2, maximum of 4 cycles of CT according to response,
(mean cycles given 2.7).

Results

Response rate (RR) after CT alone was 82% (CR 45%). After RT the CR was 601%. Four
patients remain alive and disease-free; 7 patients have did, 3 of local failure, 3 of
metastatic disease (in 1 of whom local disease control was confirmed at autopsy).
Median survival was 16 months. Grade IIVIV haeratological toxicity was seen in 5
patients, but none experienced serious sepsis. OtherwiseCTwas usualywelltolerated.

Conclusion

These prelininary results are encouraging in terms of local control and organ
preservation. The poor overaml survival, resulting from intercurrent disease and
metastases is disappointing, but anticipated for this group of poor-prognosis patients.
Recruitmentcontinues; this approach may in future merit evaluabon in controled trials.

P42         HIGH CYCLIN Dl PROTEIN EXPRESSION IN

THE CONTEXT OF P53 MUTATIONAL
STATUS RELATES TO CDDP RESISTANCE IN HUMAN

CANCER CELLS H. M. Warenius. L. A. Seabra. R. Barraclough &
P. S. Rudland. Oncology Research Unit. Department of Medicine.
The Duncan Building. Daulby Street. Liverpool L69 3GA.

Resistance of cancers to the cytotoxic effects of platinating agents
remains a major clinical problem. How ever. no consistent
mechanism to explain resistance to CDDP has vet been found.
Recently. increased expression of molecules involved in positive
signal transduction pathways such as EGF receptor tyrosine kinase.
PKC. v-H-ras and c-myc have been reported to be associated with
CDDP resistance. In addition we have observed that increased
constitutive expression of cyclin Dl protein in human in-vitro cell
lines significantly correlated with relative resistance to CDDP.
Sensitivity to CDDP is also related to p53 function in model systems
and in the clinic. We have therefore studied the expressions of
mutant p53 (mp53) protein and mutations in the entire coding
sequence (exons 2 to 11) of the p53 gene in 14 human cell lines. All
cell lines with p53 mutations showed a highly significant elev ation of
mp53 protein (p=0.003) compared to cell lines with wild-type p53. 7
lines had typical p53 mutations in the DNA binding regions one of
which was a frame shift resulting in a truncated p53 protein whilst
the other 6 were point mutations producing amino-acid substitutions.
No relationship was found between either mutations in p53 DNA or
mp53 protein levels and resistance to CDDP when only the p53 gene
was examined in relation to CDDP resistance. However when cyclin
DI protein levels were measured in the context of p53 mutational
status a highly significant correlation between these dual parameters
and CDDP resistance became apparent (r = 0.99. p = <0.000 ).

p44           BIOMARKERS FOR THE PREDICTION OF

COMPLETE CLINICAL RESPONSE TO PRIMARY
CHEMOTHERAPY IN EARLY BREAST CANCER.
C.D.Archer, S Ashley, M.Dowsett, I.E.Smith. Royal Marsden
Hospital. Fulham Road. London. SW3 6JJ.

Primary chemotherapy is now used widelv for the treatment of early

breast cancer, especially in larger tumours where the mastectomv rate
can be reduced. Its use also gives us an im vivo' measure of

chemosensitivity and a pre-treatment biopsy may be taken with which
to assess the impact of biological variables on response. We studied
58 patients who were treated with primarv chemotherapy (either
Adriamycin and Cyclophosphamide, AC, or Epirubicin,

Cyclophosphamide, and infusional 5-fluorouracil. ECF) for tumours
of at least 3cm. All patients were under 65 years and potentiallv
operable breast cancer. 21 patients had concomitant tamoxifen

therapy. Immunohistochemistry was used to asses the biological

variables on the core biopsies taken pre-chemotherapy. Oestrogen
receptor (ER) status was measured using the Dako ID5 antibodv.

bc12 with Dako 124 antibody. apoptosis by the TUNEL technique.
and proliferation with the MMB 1 antibody. 53 patients responded
(93%). 33 had a complete clinical response (CR) and 20 a partial
response (PR). Apoptosis and proliferation scores did not

significantly predict for response (p=0.76 and p=0. 14. respectively).
As expected there was a strong positive association between bc12 and
ER. Bcl2-ve patients were marginally more likely to achieve a CR

than bcl2+ve patients (74% vs. 48%. p=0.07. Fishers exact test). In
addition, when bc12 was expressed as a continuous variable. the

lower the bc12 score. the more likely a CR (p=0.04). ER-ve patients
were much more likely to achieve a CR than ER+ve (69% vs 22%.

p=0.004). In a multivariate logistic regression analysis ER predicted
for CR. Although it is well known that ER status predicts for
response to endocnrne therapy. a relationship to chemotherapy

response has been less clear. but if confirmed by further study ER
status could also be helpful in patient selection for chemotherapy.

40 Poster Presentatons

TRANSFORMING GROWTH FACTOR BETA-3
P45        MOULATION OF VENTRAL TONGUE CIRC

RHYTHM N WM. A-4 Wwdley, J.H. Scarft and C.S.
Poten CRC Deptof Epteial Biolg, Chrise Hospital & Paheson nsbte,
Maiduster M20 4BX, UK

The gastro-intestinal mucosa with it's high rate of cellular renewal
is a site of major acute toxcty from cytotoxc agents. Oral
mucositis (OM) is a visible expression of this dose-imiting effect.
There is a pronounced circadian rhythm of DNA-synthesis in oral
epithelia. in the ventral tongue the maximal rate of basal S-phase
labelling index is 30% at 04:00-05:00 and the trough <5% between
15:00 & 18:00 hours. Manipulation, with cytokkies, of stem cell
kinetics to render them more resistant to damage beore treatent
and subsequently stimulate them to prolierate more rapidly after
therapy may abroate this toxicity. Here, we have inetigated the
effect of one growth modulator on this circadian rhythm.
Transforming growth factor beta-3 (TGF-03) an inhibitor of cell
cycle progression through G1 was administered subcutaneously to
BDF1-mice at one of eight dose levels, in one of 6 schedules, in
relation to the circadian rhythm. Animals were pulsed with
bromodeoxyuridine at the time of peak or tough basal Li and
s   ifced. There is a dose dependent effect of TGF-f3 on basal
Li The timing of ss injection relative to the circadian
rhythm has a greater effect than total dose. We are investigatng
these TGF-03 protocols in an in vivo murine model of radiation
and cytotoxic drug-induced oral mucositis.

P47

AN AUDIT ON THE MANAGEMENT OF METASTATIC
RENAL CELL CANCER - SELECflON OF PATIES
FOR IEMUNO-CEMOl    ELRAFY.

Mat D, GrMffih A, Cole A, Aim D, Mam  M D. Veimdre NBS Trut,
W. A I Cauf, Wits, UK

rdm are adwied they can be of ig dirbost and possiby ce to cme.

Sedmof qi-spaft mbens for tIe tberies is inpmran

A dlical adit w  peztwnf d to iess wber plaes treud with iniam-
ciY, filed the ciaia for ilme puuls lmey to Woe from.
this km of tre -eM. Padem with ms  rl cdl c  r cms-e dhded
io td    progtic Siaps da     on ECOG ;i  -X"     (0 or 1),
dha  free ioa (>or <24  sit ), d  _ ci zfsi o  ic disa

(I or >1).  Pisial in the VoW  d xdc   po   gic pa  d   be

for           as~~~ snrvival lxnefit cmbe achieved  This
tru     15 i3is tf s   jo or jxo  cgrow qpme

Pamlsn with menl cell cance wet ideffed frm ISCO - our dmicl oerwkhiy
dtb      Thc d  w  mlysed with repido  I_ oi Casg  per year,
p, ax, sup ofuuem   I   m s      dbm free dii meerva   im of
of tic disease td It     iL

131 pi    with n      mic   cdl ca ce wtrd Iwth
m1phiesI 17%, Piezu 40%, 1.uadwa 10%, 1nke,  b    2 ad 5
Fior   l 10%,    u   uI mi y 62%  P     P PaWed f h  -

cq        hd ECOG   _c       s ofO (35%), omly o  o    bc
die (65%),      ds 2 ye Ase frie imrval (35%). Baed o them

npomc i~    ws 65% oi pokia we cmldfied good w       35%

Selac1im c~oijzias for        -,was   pMe

PATTERNS OF METASTATIC DISEASE IN PATIENTS
P46        WITH COLORECTAL LIVER METASTASES        AR

Gillams, I Taylor, JR Ledeman, PB Boulos and WR Lees.

The Colorectal Group, Middekse Hospital, Mortiner Stre, London, WIN 8AA.

OBJECT      To better elucidate patterns of disease in patients with
colorectal hepatic metas       the impact on survival and the
implications for patient selection.

METHODS The study population compised 67 patients 49M; 18F
mean age 57y (36-87) with hepatic metastes from colorectal cancer
treated with percutaneous liver laser (PLL) since 1993. Preferred
acceptance criteria for PLL are < 5 liver metastases, <5cm in
diameter. Filty one patients had disease confined to the liver at start
of PLL and 16 had extrahepatic disease; 4 had local bowel
recurrence, 4 pulmonary metasutses, 2 nodal, 3 peritoneal, and 3
some combination of the above.

RESULTS. 11/51 with hepatic disease only (22%) subsequently
developed evidence of dissemination; 4 developed pulmonary
metastases at a median of 9.5m, 3 local recurrence, 3 abdominal
lymphadenopathy and 1 widely disseminated metastases. Median
survival in the patients with extra-hepatic disease from start of PLL
was 15m and from di       is  of liver metastases 27m.  Median
survival for those with confined disease was 22m and 36m and for
those who developed extrahepatic disease 17m and 29m. Of 11
patients with pulmonary metstases, 5 had metastases elsewhere
outside the liver and a mean survival of 1I1 and 19m, 6 had only CT
detected pulmonary nodules and liver disease and are all still alive at
mean 26m.

CONCLUSIONS Although extrahepatic disease negatively impacts
survival, patients with limited (<5,<5cm) intrahepatic disease still
benefit fom PLL. In particular patients who have small CT detected
pulmonary nodules as the only manifestation of extrahepatic disease
should probably not be denied further PLL.

PERIPIERALLY INSERTED CENTRAL
P48            CATHETERS FOR AMBULATORY SFU

CHEMOTHWERAPY: A NURSE LED CLINIC.
VAston, C.Barker, A. Brewster, T.S. Maugan     Cardiff.U.K.
Patients with advanced cokxect can   requiring palative
c    d~txAI~rapy piefer to receive as much of their treatment as

possille m the outpatiat setting. 5Fuorouracil remains one
of the mainstay trments fur this group of patients.

Continuous infusio of 5FU cxpose the malignant cells to

constant levels of cytotoxics This method has been shown to
result in high response rates (30%) with low levels of toxicit.

It is currenthl being investigated as one arm of the MRC CR06

In 1997 at Veliixre NHS Trust, a weekl peripheraih inserted

central cathete (PICC) clinic was developed, primaril- for patients
to receive ho ambul     5Fluorouracil The clinic is led bt the
two specialis nurses and between 7-8 PICCs are currently placed
each we. To support this initiatie a wckl punp change clinic
has also been developed, wher patients receive weeky assessment
by the c    ap    specialist nurse and are fillh re-iewed by the
cnsultant once a mnth ProIuss  h  produced audio cassettes

giving inr m on on specific cmnherapy regimens have been well
receiv    by patients.

Between January 1996 and Janay 1997, 131 PICCs were placed,

of which 53 (40?/%) wer specifically for the advanced colorectal group
to receive ambulatory 5FU. The compliaion rate of the PICC lines
placed remains low. One line only was removed due to infction,

tdxre have bee 6 (4/) record  local site infection (usually during
the Nadir). All resolved spoanusy or with oral antfiowtics.

There have been 9 psodes (7%) of tdunbosis(usuallh when the
PICC migrated out of the SVC) The other mai complcation has

bee n-hAnica1 phlebitis 7 (5 %)episodes, all but one were resohed
with heat treat  and/or anti-inflammatory nmeication

As a result of these new services,  le financial cost benefits
have been made, as well as improving tIe quality of lif of patients

and teir 6milies.

Poster Presentations 41

Lung Cancer Referal Patterns - A Pilot Study

P49         SM Crwford' and P Mdi2 for the Yorks Lung
Car~ Audit Gop 'Aiedale NHS Trust, BD20 6TD 2Northen &
Yorks Cance Registy & Ift        Svi

The So    w of kng can     in the UK are inferior to those in some
odh  Euroan cxAtries. We decided to omxc a detailed study of
te   patients to         the process of          . In order to

F th o    i 0  a pib t udy has bo  codMAXA

50 patiats were i      i       the Cancer Registry who were
diagnosed in 1992 and managed withen the Natio l Health Service in
the old Yorkhire Region  Comparison of this s   with the Registry
dataliase shows a smg excess of m a piats (74% verus 65.5A).
The Gedneral Pr        (GP)   e of 46    *   were avmsiland
the  uspital       fom 43. No notes wer   aced on 2 and one was
fomnd to hae bern daosae    neoropsy.

In 22 caes (cl      ptw), the GP had considered a         of
lung c      as a poslblity, a  t x-ray was  rmed and a rerral
made to a hospital c nmilta, in 2 ce  is was a gea phl

This reiferal was   a noian of 1.5 days ae the x-ray report was
rceived by the GP; the m       interval was 45 days. The median
time firm  _riIestao  to refIrral was 20.5 days; max 137 days. In 11
cas  (non-classkcal pathway) the diagoss was amt co ed by the
GP amd the patiew was refiered for ieigatI of symptoms to a wide
vuiay of seialists. 13 we admitted to hospital as oe        .
Histology was obtained in 90% of classical  c  72.7%  of non-
daslkW amd 50% of those     ing as ae e       ne    The overall
listolog rate was 74.4%. Bronchoscopy was   med in 33 (76.7%)

paIes am ni      X m4 - i 3 (7%)- 7   1aet up     ntofsl

with acrative ime  in 3 (7%).   n       Y was a         d to 5
pi     and pa       r       a   witlt other -ratis to 15
(34.9%). Overall the ndimi iterval beween the first hospital vis mxd
a decision   t tb-ret  being madetwas 31.5 days with a roe- firm
0 to 175 days. For classical patieus, the median was 21 days; max 79.

CAN INTERLEUKIN-2 IMMEDIATELY AFITER
P51         HIGH DOSE CHEMOTHERAPY WITH

PERIPHERAL STEM CEL L TRANSPLANTATION
IN PATIENTS WITH LYMPHOMA BE A SAFE AND EFFECIIVE
WAY OF CAUSING A GRAFT-VERSUS-LYMPHOMA EFFECT?
L S DobsonI, M Duggan-Keen3, J A Radford2, P L Stern3, G R
Morgenstern2, J H Scarffe2, and D Crowther2. Yorkshire Cancer
Research Dept. of Clinical Oncology, Weston Park Hospital, Sheffield,
UK. 2CRC Dept. Medical Oncology, University of Manchester,
Christie Hospital NHS Trust, Manchester. 3Paterson Institute of
Cancer Research, Manchester, UK

Obiectives: To give IL-2 from day of stem cell return for eight days
after high dose chemotherapy (Busulphan, Cyclophosphamide) in an
attempt to safely obtain a graft versus lymphoma effect.

Methcds: Five patients received IL-2, 4 x 105;6IU/m2/day s.c. BD
while a further five patients were monitored (the control group).
Toxicity was assessed by CTC criteria, observation charts, serum
biochemistry. Haematological recovery was determined by full blood
count recovery and blood product requirements. Immunology was
assessed by FACS analysis using antibodies against CD16, CD56,
CD3, CD20, CD4, CD8 and CD25. Function was assessed with in
vitro NK and LAK assays.

Results: Four patients completed IL-2 therapy, one patient withdrew
with psychiatnc problems. Three patients exhibited a widespread non-
specific rash, three expenenced grade III/IV mucositis and two had a
weight gain. The mean number of days of pyrexia increased (10 v 5.8
days) despite no increase in number of septic episodes, and the length
of hospital stay increased from a median of 13 days to 17 days. Mean
blood product requirements also increased (7U pack cells, 17U
platelets v. 3U and 1lU). Two patients in each group had deranged
LFTs whilst only in the IL2 group was renal impairment seen in two
patients (days 7-17). Early elevation in NK cells was observed (at day
10- 14 v. 19-28) but this was not prolonged. No fall in CD3+ cells was
seen immediately after PBSCT and this was due to an increase in

CD3+ CD4+ cells. This delayed reversal of the CD4:CD8 ratio to
days 19-28 (v day 10-14). No increased cytotoxicity was seen.

Conclusions: Whilst numbers entered into the study were small,
toxicity was significant.

TIME TO CT DETECTION OF 2-'D HEPATIC

P50        METASTASES AFTER RESECTION OF COLORECTAL

CANCER? AR Gillams, I Taylor, JR Lederman, PB Boulos,
WR Lees. The Colorecal Group, Mfiddlesex HospitaL Morimer Stree London

OBJECT Screening for colorectal liver metastases has been
controversial, but new therapeutic techniques and improved
detection with spiral CT suggests screening should be revisited.
Our aim was to determine the time to CT detection of new liver
metastases in a cohort of patients with known colorectal liver
metastases undergoing percutaneous liver tumour ablation (PLTA).

METHODS Sixty-two patients, 47M; 15F, mean age 57y (36-87)
underwent serial CT scans every 2-3 months for post PLTA
surveillance. First liver involvement was diagnosed at bowel
resection in 36/6. Post operative diagnosis was made at median
1lOm (4-43) post primary resection; following a nse in routine CEA
in 6/62 and on imaging surveillance in 20/62. The post PLTA CT
scans were retrospectively reviewed for the development of NEW
metastatic deposits in previously normal liver.

RESULTS During     PLTA   follow-up  38i62  (6100) patients
developed new liver metastases at median 13m (3-49) post primary
resection. 23 of these 38 (61%,/0) developed further new metastases
at median 18m (7-51), 7/23 further lesions at 25m (10-55) and 3 7
subsequently. Mean CT follow-up was 14t- -9m. Of a total of 71
observed new metastatic events, 50% occurred within 16m, 72% by
24 m, 86% by 36 m and 94% by 48m.

CONCLUSION Serial CT analysis of patients with known limited
liver metastases showed new events occurred in the majority by 2
years, but 28%/ developed new metastases after 2 years. This may
have   implications   for   those   considering  screening.

P52        CYCLOSPORIN A - CAN IT BE EFFECTIVELY

GIVEN FROM THE DAY OF STEM CELL RETURN
IN PATIENTS WITH POOR-PROGNOSIS NON-
HODGKIN'S LYMPHOMA UNDERGOING HIGH DOSE
CHEMOTHERAPY WITH PERIPHERAL STEM CELL
TRANSPLANTATION. L S Dobson1, M Duggan-Keen3, J A
Radford2, P L Stern3, G R Morgenstern2, H J Scarffe2, D Crowther2.
1Yorkshire Cancer Research Dept. Clinical Oncology, Weston Park
Hospital, Sheffield UK  2CRC Dept Medical Oncology, University of
Manchester, Christie Hospital NHS Trust, Manchester, UK. 3Patcrs n
Institute of Cancer Research, Manchester, UK.

Objectives: Cyclosporin A (CsA) was given to patients undergoing
high dose chemotherapy with Busulphan and Cyclophosphamide in an
attempt to obtain an autologous graft-versus-host reaction (aGVHR).
Treatment effects were monitored and compared with a control group.

Methods: CsA (5mg/kg/day) was given orally from day 0 to day 28.
Five patients were entered into each group. Toxicity was assessed bv
CTC cterita, observation charts, serum biochemistry. Haematological
recovery was assessed by full blood count recovery and blood product
requirements.  Immunology was assessed by FACS analysis of
lymphocytes using antibodies against CD16, CD56, CD3, CD2O, CD4,
CD8 and CD25. Function was assessed with NK and LAK assays.
Any rash was to be biopsied.

Results: Four patients completed CsA therapy, one patient withdrew
due to excessive nausea. No significant toxicity was expenenced.
One patient developed a palmar rash on day 8 which lasted 36 hours,
unfortunately a biopsy was refused. There was no delay in
haematological recovery or an increase in blood product requirements.
The proportion of NK cells rose on day 19 as expected but continued
to be elevated until day 47. The proportion of CD3+ cells
unexpectantly rose on day 10-14 (mean 91.5% compared with 36%),
the majority being CD3+, CD8+ cells. No increased cytotoxicity was
seen.

Conclusions: Numbers entered into the study were small. The
treatment did not have anv adverse toxicity, however, it failed to
produce a confirmed aGVHR

42 Poster Presentabons

SOFT TISSUE SARCOMAS. A NEED FOR
P53             ORGANISING THE PATTERN OF

REFERRAL

LM El-Helw', PC Lorigan', J Goepel2, MH Robinson'.

'Yorkshire Cancer Research Department of Clinical Oncology,
Weston Park Hospital, Sheffield S10 2SJ.

2Dept of Pathology, Royal Hallamshire Hospital, Sheffield.

Objective: To assess the quality of treatment of soft tissue sarcoma
patients referred to Weston Park Hospital since 1992.

Methods: Prospectiv e data has been collected for all new patients seen
at the sarcoma clinic between January 1992 and June 1997.

Results:

Three hundred and fifty patients were registered with a diagnosis of
sarcoma in North Trent during this period. 208 patients with soft tissue
sarcoma; 97 males (46.6%), 111 females (53.4%) were seen in the
multidisciplinary clinic. 151 (72.6%) of these were new cases. The
median age was 60 years. Most referrals were made by a general
surgeon (66 patients or 31.7%) or an oncologist (56 patients or 26.9%).
47 (22.6%) patients were referred for definitive treatment before
surgery and 67 (32.2%) after surgery, the remainder were seen with
local recurrence (17.8%), metastasis (7.7%), both local recurrence and
metastasis (2.4%) and 36 (17.3%) for follow up. 35 out of 67 patients
(52.2%) referred after primary surgery have required second pimary
operation. Overall 59% patients in North Trent with a new diagnosis of
sarcoma were referred to the sarcoma clinic. Only 20% were seen
before primary treatment. How ever, it appears that there is
improvement in the pattern of referral after January 1992' as 37
patients out of 151 (24%) with soft tissue sarcona were referred to the
sarcoma clinic before primary treatment, while only 2 patients (4%)
w ere referred before primary treatment prior to January 1992. There is
also improvement of the treatment pattern as 36.8% of patients were
treated with a multidisciplinary approach in 1992 compared with
71.4% in 1997.

Conclusion:

The introduction of a multidisciplinary clinic has improved the initial
treatment of sarcomas since its inception in 1992.

P55          HIGH DOSE CHEMOTHERAPY FOR

SOFT TISSUE EWING'S SARCOMAS.

R.E. Hough, S.Browne, L.M. El-Helw,
R.E.Coleman, M.H. Robinson, P.C.Lorigan. Yorkshire Cancer
Research Department Of Clinical Oncology, Weston Park Hospital,
Sheffield. SlO 2JS.

Soft tissue Ewing's sarcoma, peripheral neuroectodermal tumours
(PNET) and Askin's turnours are small round blue cell tumours which
characteristically have a reciprocal translocation between chromosome
11 and 22. As with other rare tumours, clinical trial data on the
optimal management of these patients is limited. We review the
results of 6 patients treated with conventional chemotherapy and
compare these to 9 patients treated with standard induction regimens
followed by high dose chemotherapy (HDC).

6 patients (1 female, 5 male) received standard chemotherapy with
IVAD (or similar regimen). Mean age at presentation was 23 years
(range 16-33 years). 2 had localised disease, 2 locally advanced and 2
metastatic disease. Additional radical radiotherapy was given to 5
patients and surgery was performed in the other. 4 patients have died
at a median time of 284 days. Survival in the 2 remaining patients is
1947 and 2006 days.

9 patients received IVAD (7 patients) or EVAIA (2 patients) induction
chemotherapy. Mean age at presentation was 26 years (range 17-39
years). 3 had locally advanced disease with the remainder having
metastatic disease. Complete response (CR) was achieved in 2
patients and a good partial response (PR) was seen in all others. HDC
in all patients was with Etoposide, Carboplatin and Melphalan and a
further 3 CR were achieved. Additional radical radiotherapy was
given to 6 patients. 6 patients have died after a median interval of 421
days. 3 patients are alive and in remission at 417, 666 and 884 days.

Soft tissue Ewing's sarcoma often responds to conventional
chemotherapy but tends to relapse, and the long term prognosis is
poor. HDC may offer some survival advantage but multi-centre
clinical trials of this and newer strategies will be vital in improving the
outlook for these patients.

p54           IS COMPUTED TOMOGRAPHY

NECESSARY TO EVALUATE PULMONARY
METASTASES IN PATIENTS WITH
PERSISTENT TROPIHOBLASTIC DISEASE? A M Gillespie, R E
Coleman, B W Hancock, Trophoblastic Disease Centre, Yorkshire
Cancer Research Dept. Clinical Oncology, Weston Park Hospital,
Sheffield. UK.

The aim of this study was to compare the use of chest radiography
(CXR) and computed tomography (CT), in the prognostic risk
assessment of patients with persistent trophoblastic disease (PTD).

Methods: Patients treated for PTD with chest metastases between
1987 and 1996 were identified and the original case records were
reviewed to determine the risk score attributed to pulmonary
metastases using CXR The Charing Cross prognostic scoring system
gives 1 point for 1-4 pulmonary metastases, 2 points for 4-8 and 6
points for greater than 8. At the Sheffield Centre patients with an
overall nsk score of 7 or less are classified low risk' for the purposes
of defining appropriate chemotherapy.

Results: 69 patients were identified with pulmonary metastases.
CXR and CT were performed in 63 cases and CT only in the
remaining 6 cases. Of the 63 cases where both investigations were
performed, 36 patients had metastases detected by both modalities
and 27 by CT only. When both investigations were positive the CT
scan tended to be more sensitive and would ha'e increased the
prognostic risk score in 19/36 cases, changing the risk group from
low to high in 7/19 cases. In the 27 cases where CT only was positive
the prognostic risk group would hase altered in 4 cases. In onl  J 2111
cases where the risk group would ha'e changed by using CT was
sal age chemotherapy actually required.

Conclusion: This study shows that CT is a more sensitive imaging
technique than CXR for detecting pulmonary metastases in PTD.
Howe'er, using the Charing Cross prognostic scoring system ,CXR is
an adequate investigation for directing patient care. This information
is particularly useful to clinicans in developing countries without
ready access to CT imaging.

56          TREOSULPHAN MONOTHERAPY FOR
P5            MEITASTATIC BREAST CANCER - A

PHASE II STUDY. LS. Dobsonl. S. Clive2,
L Daw son2, A. Anderson2, RC.F. Leonard2, I. Kunkler2 and R.E

Coleman1 iYCR Dept. of Clinical Oncology, Weston Park Hospital,
Sheffield, 2Dept of Clinical Oncology, Western General Hospital,
Edinburgh.

Treosulphan is an epoxide-type alkylating agent which has
been previously used in an oral formulation, particularly in the
treatment of ovarian cancer. An intravenous formulation is now
asailable and the aim of this study was to determine the rate of
response and toxicity to this therapy in patients with metastatic breast
cancer. 8g/m2 was given by a short intravenous infusion on a four
weekly basis with planned dose escalation.

Fifty five women were recruited, 33 from Edinburgh and 22 in
Sheffield. In 38 women this was first line therapy for metastatic
disease. The median interval from diagnosis to relapse was 6.5 years
and the median age at treatment 68 (range 43-s8). Forty five women
had previously received radiotherapy, 44% in an adjuvant setting.

The median number of courses received was three and only one
patient had dose escalation. In twelve patients there was a dose
reduction, seven of these had a 25% dose reduction and five a 50%
reduction. This was predominantly for delayed haematological
recovery. Toxicity was mild, the most frequent was grade 1 nausea.
The overall response rate was 13% (13.2S% first line, 11.8% second
line). Both CRs occurred with first line treatment Treosulphan has
the potential of being included in dose intensive regimens since it is an
alkylating agent with little second organ toxicity. Because of the
moderate activity and modest subjective toxicity treosulphan is a
potential palliative treatment for poor performance status or elderly
patients.

Poster Presentations 43

P57

SALVAGE WITH CHEMOTHERAPY AND RADICAL
RADIOTHERAPY OF CARCINOMA OF THE CERVIX

INiTIALLY TREATED BY SURGERY WITH CURATIVE

INTENT. B. Seddon, N. Lodge, P. Blake. Department of
Radiotherapy, Royal Marsden Hospital, London.

Early stage (FIGO IB and IIA) carcinoma of the cervix
may be treated by surgery or radical radiotherapy,
surgery is often chosen as the primary treatment
modality, and relapse after surgery occurs in up to
15%. We present a series of sixteen consecutive cases of
patients treated between 1985 and 1996 for relapse of
stage I or IIA carcinoma of the cervix after surgery
with radical intent, salvaged by use of chemotherapy
and radical radiotherapy. Patients were all treated with
external beam radiotherapy, and five patients
additionally received brachytherapy. Nine patients
received three or four cycles of PMB chemotherapy
prior to radiotherapy. Overall survival for the group
was 62.5%, with a relapse free survival (crude salvage
rate) of 44%, after a median time to recurrence of 28
months. Local control was achieved in 50% of patients,
and was essential for long term survival. Local control
was best achieved by radiotherapy doses in excess of
60 Gy; the use of brachytherapy appeared to be
important for reaching these higher doses.
Chemotherapy did not appear to improve survival, and
it's addition to external beam radiotherapy and
brachytherapy probably increased major morbidity.

P59        EXTENDING THE ROLE OF THE THERAPY

RADIOGRAPHER: A REPORT ON BEHALF OF THE

"SIONGROUP OF THE COLLEGE OF RADIOGRAPHERS.

Charlotte Beardmore, Gill Ross. Academic Radiotherapy
Unit,Royal Marsden Hospital,London

The Calman-Hine model of Cancer care requires that services
will become more patient-centred and cost-effective.Given
resource constraints, these aims must be achieved largely by
redefining mnodels of care delivery, induding roles of heafth
care professionals in the multidiisiplinary team. This requires
an evaluaton of existing expertise and atitudes to change in
specific professional groups. The aim of this study was to
conduct a needs assessment study of the current status of
therapy radiographers, in order to develop an understanding
of the  capacity for expansion  of teir role in the
multidisciplinary cancer team. Methods: A strutured
quesonaire evaluating profesional views and perceptons of
role extension was sent to all UK therapy radiographers; a
strutured questonaire to a cross section of allied healfth care
professionalsfocus group interviews with selected groups of
terapy radiographers currently involved in role development,
and managers of therapy departmnts. Results: Full data
sets were obtained for current practce in radiographer-led
clinics,  radiotherapy  treatment  skills,  er    apy
administration, audit, clinical trial co-ordination and patent
counselling. 46% of 793 radiographer respondents indicated
that they had existing skiDs in patient care and team-related
skills that were underutilised. Only 20% were regularly
responsible for pre/post or on-treatment pabent review,while
over 50% wshed to develop this role.

Conclusions: Therapy radiographers constitute a highly
skilled workforce , with the motivation and capacity to
undertake a significantly extended role in health care delivery
in the clinical oncology team of the fiture.

P58             Stereotactic Multiple Arc Radiotherapy in

Meningiomas Invoh-ing the Cavernous Sinus.
ASibtain and P.N.Plowman

DeptofRadhapy,StBar        Baolomew'sHosptal, London ECIM7BE

Twenty-eight patients with meningioma (27 bem'gn, 1 malignant)
involving the cavernous sinus have been referred to the radiosurgical
team  at StBartholomew's Hospital between Februars   1989 and
February 1998. The median age wmas 44 3 years and the female: male
ratio was 22:6. Eleven patients presented with decreased vision,
eight had altered ocular movements and other cranial nerves were
affected in seven patients. Three patients presented with headaches
and one each with fits, unsteadv gait and secondars amenorrhoea
Tumour size at referral was < 3cm in five patients, 3-5 cm in 15
patients and > 5 cm im 8 patients. Nine patients were treated without
pnor surgery, fourteen had had one excision, four had had two
excisions and one had had three.

Nine patients received Stereotactic Multiple Arc Radiotherapy
(SMART, x-knife surgery) as a single fraction, one had fractionated
SMART, and five received conventional fractionated radiotherapy
(CFRT) to the skull base Three patients were treated with both
techniques.

Large size, proximity to optic nerves or chiasm (with or wlthout
some visual loss and/or other sensitive structures influenced the
choice of treatment Small lesions abutting the visual pathways lend
themselves to frationated SMART.

Of patients presenting for RT with tumours <3cm, 8/14 received
SMART versus 3/14 receiving CFRT, and 3 14 receiving both. Of
patients with twmours >3cm, 2/14 received SMART versus 12/14
receiving CFRT. Median follow up <I8months.no relapse to date.

The indications for different radiotherapy strategies are discussed
These patients are best managed in centres with radiosurgerv and
conventional radiotherapy facilities

P60            TMLE: CHEMORADIATION USING

CISPLATIN AND 5 FLUOROURACIL
IN PATIENTS WITH ANAL
CARCINOMA
T.RlHolt, RP.Crellin, J.BJ.Fozard, R.Talbot.

Department of Clinical Oncology, Poole Hospital, Poole, BH15 6JB,

Department of Colorectal Surgery, Poole Hospital and Bournemouth
Hospital

Purpoe: Combined radiotherapy and chemoradiotherapy is the treatment
of choice for locally advanced anal carcinoma as shown by studies

comparing concomitant use of radiotherapy and Mitomycin (MMC) plus 5
Fluorouracil (5 FU) to radiotherapy alone. It has been suggested that

substituting Cisplatin (CDDP) for MMC may improve locoregional control

rates with acceptable toxicity. This study reviews our experience using high
dose radiation, CDDP and 5 FU for treatment of anal carcinoma.

Method: Eleven patients with a mean age of 54.6 and TNM staging of

T2(4); T3(3); T4(3) and N0(10); N1(1) were treated. Ten had anal canal

tumours,one had an anal margin tumour. Patients were given 45 cGy in 25
Fractions of 1.8 cGy over 5 weeks. Weeks 1 and 5 were accompanied by 4
days of continuous infusion 5 FU 1 g/mn and CDDP 25 mg/n? given over 4
hours on days 1 through to 4. Patients were then rested for an average 18.6
days to allow the brisk acute reaction to settle before receiving a reduced
volume Phase II of radiation 15-20 cGy given in 6-8 Fractions. Aserage
time for follow-up was 15.4 months, range 3-27 months.

Results: Complete response was achieved in 10/11 (91%) patients by 4

months following tieatment. One patient had relapsed locally with distant

metastases 3 months following treatment. No patients died. One patient had
a defunctioning colostomy prior to treatment which was reversed 2 months
following treatment. One patient experienced Grade 4 early toxicity and 2

patients Grade 4 late toxicity. 91% of patients are alive with no evidence of
disease and in all, the anus and its function has been preserved.

Conclusion: Treatment with this regime seems to offer equivalent if not
better locoregional control than MMC/5 FU and radiotherapv with

acceptable toxicity. However follow up in this group of patients is short and
the results must be viewed with caution.

44 Poster Presentations

Pe ^~CIRCULTING COLORECTAL CANCER CEI I
P61                  MUSTERS DISPLAY           Hk-ROGENEITY         IN

EXPRESSING CEA AND CK20.

RQ Wharto, SK Jonas, Z Khan, C Glover, M Henry, TG Allen-Mersh, Dept of
Surgey, Imperial College Sch of Medicia, Chelsea & Westminster Hospital,
Loodon.

BnekgW~adi.

Cell mutations in solid tumours produce subpopulations which have differing
prpesies. We have previously demonstrated that RT-PCR can be used to detect
cdrcuinag tiMmnxin cells in patients with colorectal cancer by identifying mRNA
coding for C   anmtwy-onic antigen (CEA) To detenmine wheter circulig
t-m      cls are also hegnu      we examied the prevalence of tumour cell
Hecti    u    two mRNA markers: CEA and cytokeratin 20 (CK20).
Metods:

Three fourteen ml samples of peripheral venous blood were obtained from 53
colorectal camcer paients at 1 minute interval& After the addition of erythrocvte
lysis buffer, total RNA was extracted by conventional methods from the remaining
cells and subjected to RT- PCR in separate reactions using either CEA or CK2O
cDNA primers. The PCR products were run on electrophoretic gels and the product
confirmed by Southern blotting using an oligoprobe. 43 patients without known
carnoma were used as controls.

There was hetrgeneity of mRNA expression in 61/114 samples (28/53 patients).
We conclude dsta tere was variation in the expression of antigens by circulating
taunor cells from coloretal cance patients and that the true prevalence of
crlating coo    tal cancer cells was higher than estimated by RT-PCR based on
sing  anigcn mRNA.

CMB401 - A NEW AGENT FR IMMUNOTHERAFY
P63       OF EPIELIAL OVARLAN CANCER: IMMUNE

REONSE AND PHARMACOKIETCS

TJA       K AL (Ma, RE Cdak~a, J. Owz, L S1mn3, S.
Soqbcw, A. Fiam, K 9wi3 & S.Y.Cbn.'.lDq CQic  ?

ciy  il, Notil, UXe; 2YC Dqpt Omik IAk, Waim Pak

HI    S    UK; 3C e_  Ts  L ad, Skcomh UK.

1     :d ctmCIBZ401 is a Tc R  eR

evo -tofor i(ume         O   cncel   a

~~~o f a~~~~~~~o n~~~~~ y  -~-!:  hm m an o

is a    Of da  yWuW a               poemorphicy,

1 i            by     wh

0  : To ~~~asn the _i     md     _

fCMBz401 fiowv a     eno  da_
it  a    I

IN. I h 34 pus with rawrent EOC rec1ed up to 4 cycl of
35~ va        CTM"01pe-doe followed by 2-16 mgn of

imuevais of four hwedk. This  schekile- was
dialme to  ci -lOr PEK  , ahw moSm Altsue

lion Bb~Blod                 nk n c irafm   Of
CNIB401, tow hCfKi    g  wPEM  Ed u   MOI

and uami-calidmomnicin

Reob: Levels of cireating PEM declined  m
each  j u   T m x z   neaitralion      was

onaisteit abroughout the tr ia t cycles with gre   n th e
wa.ivel Ordose A _y        a X do by
fife of CMhOP4  a 36.3   devaEndU1 26.8). No

Hid any         rewoll n Ad only 3  hs oa

* I o w ~ t e d   e d aisi atio n  o f  1 O alile  it's  thd  m M9pF t.
go zmw      _   o ofCN  01 ISma bk it's  Lou
use min)

P62

Low dose Folinic acid with bolus and infusional 5 Fluorouracil
chemothapy in advanced colorectal carcinomas.

N. Shah, P.Hosldn, M. Saunders, R. Novell, A. Makris
Mount Vernon Hospital, Nortwood, UK

Aim: To assess    e activty of tow dose Folinic acid (FA) with bolus and infutsonal 5
Fkrouracil (5FU) in advaIced or metstatc cokets   canoma

Patients and Mhods: 20 patents (11M: 9F) with a median age of 66 years (range 51-80
years) with bocaly advanced or metastabc colorectal carcnona were studed. Treaiment
consisted of 6 cycles of d ot       uskig a modified De Graniont regide [1] with the
dose of Foki acid reduced from 200 mg/rn tI 20 mg/rn2. This was a regimen of IV Folnic
acid 20 mg/n2 as a slow bokis Bkoed by bokus 5-FU 400 mgihn2 and 22-hour infusion 5-FU
600 mgn2 for 2 consecutive days every 2 weeks. Responses were aessed with respect to
improvement in patients sryptos or an obctive change in biochemical or imaged
par ai eters.

Results: 17 patients had evaluable disease for objective response. 3 patents did not have
evaluable disease but were assessed for toxicity to chemotheapy.

4 patients (23%) had a partial (>50% objectwe regression) response (3 liver, 1 bcal) and 7
patients (41%) stowed no change in their dsase status. 6 patients (36%) progressed
through dchmthepy. No complete responses were documented. A median survival of
10.6 months from the onset of paWative chemothrapy was observed. 25% of patents
remaned alive at 2 years.

Toxicity: 17 patients completed a ful course of chemoterapy without dose delays. 11
patients reported diarrhoea, of which 3 required regulat antidiarrheal medication. 15
patients reported nausea, with 3 patients mantained on regular antiemetics. There were no
irtkdences of neutropaenic   or toxcity related admisseons. 1 patient required titatinent
for te palmar plank syixome.

Conclusion: This regim  dem   srecoparable responses to prospectively evaluated
pallte    eotheapy regimens, including the De Grarnont reien with Folinic acid at a
dose of 200 mgin2. it merits further evcal on in view of is acepble toxicity profie, ease
Of a   istation and cost benefit due to the bwer dose of Foinic acid.

1.      De Graiont, A., et at, Hh-dose faduic acid and 5-furac       bolus and

contnuous ritzs    in advanced cdorecta cancer European Journal of Cancer &
Clinical Oncology, 1988. 24(9): p. 1499-503.

USE OF PERIPHERALLY INSERTED CENTRAL
P64       CATHETER (PICC) FOR ADMINISTRATION OF

INFUSIONAL 5-FU CHEMOTHERAPY. A. Lawton, S.

Affleck, L Nokes, T. J. Iveson, CRC Wessex Medical Oncolgy Unit, Royal
South Hants Hospital, Southampton, S014 QYG.

Many current treatments for gastro-intestinal malignancy utilise
infusional 5-FU chemotherapy. Traditionally this has been
administered via a tunnelled central venous line inserted by

either a radiologist or an anaesthetist. Recently PICC lines have
become available and we report our experience of these lines

inserted by nursing staff for the administration of infusional 5-FU
chemotherapy.

Between October 1996 and January 1998 nineteen patients who
were due to receive infusional 5-FU chemotherapy for gastro-
intestinal malignancies have had a PICC line inserted by the

nursing staff. Following the insertion of the line all patients were
commenced on prophylactic warfarin 1 mg once a day. These

lines were left in situ for the duration of chemotherapy which was
between 3 and 6 months. Fifteen patients (79%) completed
their infusion with no problems. Three patients (16%) had to

have the line removed because of venous thrombosis. One line
fell out Two patients (1 1 %) required antibiotics for a line
infection but did not require removal of the line.

This data shows that PICC lines are a safe and reliable
alternative to a tunnelled central venous catheter for the
administration of infusional 5-FU chemotherapy. The

complications from these lines appear similar to a tunnelled line
but avoid the risk of pneuothorax when the line is inserted.

Pbster Preswvtat   45

PHASE I STUDIES OF 'TOMUDEX'
P65          (RALTITREXED) WmIH PELVIC

RADIOTHERAPY (RT) FOR ADJUVANT

TREATMENT IN PATIENTS (PT) WITH OPERABLE RECTAL

CANCER (ORC) & RECURRENT/INOPERABLE RECTAL CANCER
(lRC) N Dbmwod *R James, C Vernon. C Pwnell and P Price.

Depwtmeut of Clinical Oncology, H iith Hospital, London and
*Clistie Hositl, Mancheste, UK

RT is used in the management of ORC adjuvansly to resuce the risk of local
recire   & possibly improve sunvival & for IRC for  1pltom and

tnour control. The addition of ceohp to such RT may be

beneficl, but when combined with pelvic RT, toxicity can be increasa
The first Phase 1 study aimed to deermine a irconunended dose of

'Tomudex' (A quinane folate anaogue acting as a pure thymidiae

synthase inhibitor) for use with juvant post-operative pelvic RT in ORC.
Pts with histologically confirmed completely excised, Duke-s B or C ORC
Vwre rccnitedL RT dclivered 50.4 GY. in 1.3 Gv daily fractions, to the

postei pelis via 3 fid. Escating dose kleves of 'Tonudex %erc:2

mg/i;, 2.6 mg/mn' 3mg/mF. 'Tomudex was given on days I and 22 one hour
prior to RT. Of 3 pts recruited at the first dose Level (2mg/m-) 2 pts

experienced dose limiting toxicitics (DLT). one grade 4 diarrhoa (pt
rqird withdrawal from RT at 35Gy) & one developed transient

asvmptomatic grade 3 transaminitis. Five frwher ps recruited a dose level
I showed no further DLTs. The 3 pts recuited at dose level 2 (2.6mg/m:)
s%'Ce wit  DLTs. All 3 pts at dose level 3 (3mg/in-) had DLTs after I

cvyck grade 3 kucopenia (2 pts), grade 3 diarrhoea and grade 2 kucopenia
(Ipt). None vxre able to recive the second dose of Toamudcx . The

recommended dose of 'Tomudex wen combined with adjuvant RT in ORC
is likely to be 2.6 mg/m2. An additional 6 sbjects are being recruitd

at this dose level. Adjuvant postoperative RT combined with 'Tomudex'
results in increased pchic toxcityh and  and Tomudex dosc

reduction is required. This   appeas to be of a similar magnitude
to the 5FU dose reduction needed hen 5RFU is combined With RT. Thc

second Phase I study has now startcd to dcefin the optimal dose of -Tomudex

plus RT in pt with IRC. RT delivered 50 Gyin 2Gv dailv fractions & the esca-

lating dose of 'Tomudex was sinular toTral 1. 4 pts so far have benrecrited
at dose level I (2mg/m2). No DLT have been reported. The trial continues
Tomudex' is a trademark; the property of Zeneca Lid

P67          HIGH DOSE CONSOLIDATION THERAPY WITH
PERIIERAL BLOOD STEM CEIL SUPPORT (PBSC) AS INITIAL
TREAMENT FOR ADVANCE) EPFTHEIJAL OVARIAN CANCER
JA Lelermann', P Murray2, K Denby', L King', AC Silverstone', AL
Jones3, G Prentice3, W Reid3, AH Goldstone'. Oncology, Haematology
and Gyncoogy Depts., UCL Hospitals', London WIN 8AA, Essex
Coumty HosPitaf', Colchester and Royal Free Hospital3, London NW3.

We have conducted a phase I/II study to evaluate the use of
high dose chemotherapy (HDC) as consolidation therapy for
patients (pts.) who have responded to platinum-based
chemotherapy with normalisation of CA125. 16 pts. (41-60 med
49 yrs) with FIGO Ell (11) or IV (5) were treated between 8/94
and 9/97. 7/8 pts. who had 'second-look' surgery prior to
consolidation HDC had active tumour removed. 15/16 pts.
underwent HDC with PBSC support; one developed
neutropenic     sepsis    and    renal    impairment      during
cyclophosphamide priming. The HDC regimen was
carboplatin AUC 18-30, cyclophosphamide 120 mg/kg and
etoposide 900-1800 mg/m2 divided into 3 doses over 5 days. 1
pt. died with intestinal obstruction during the procedure.
Recovery of neutrophils (>1.0) day (D)+ 9-15 (med.11), platelets
(>50) D+ 11-34 (med. 16). Diarrhoea was the main non-
haematological toxicity attributed    to the    highest dose of
etoposide   and   carboplatin. Etoposide    1200  mg/m2       and
carboplatin AUC 24 produced acceptable toxicity. 9/16 pts. have
relapsed and 6 have died. 10/16 (62.5%) pts. are alive 10-50
months (med. 25) after diagnosis. HDC has acceptable toxicity
but needs to be evaluated prospectively in a randomised trial
which will start in March 1998.

P66           GAMMA LINOLENIC ACID WITH TAMOXIFEN

AS PRIMARY TIERAPY IN BREAST CANCER
F.S.KennyI S.Pinder2 1.O.Ellis2 RP.Bayce3 JF.RRobrtson' Dept of Surgery
and Pathology2 Nottingham City Hospital; Scota Phar ucals Stirimg'

Aim. Gamma linolenic acid (GLA) has been proposed as a valuable new
cancer treatment having selective anti-tumour properties with negligible
systemic toxicity. This is the first study to investigate the anti-cancer effects of
GLA combined with primary endocrine therapy.

Methods. 38 patients with elderly primary (n=20), locally advanced (n=14) or
metastatic (n=4) breast cancer consented to take 8 capsules of oral GLA/day
(total=2.8gns) in addition to primary tarnoxifen. Clinical response (by UICC
criteria) was compared with a matched control group on tamoxifen (T) alone
(n=47). Serial tumnour core-cut biopsies were taken for assessment of changes in
oestrogen receptor (ER) expression during treatment

Results. The T+GLA cases achieved significantly faster clinical response
(CR/PR v-s. SD) than tamoxifen alone (see Table). T+GLA cases with larger fall
in ER at 6 wk biopsy had significantly better early response (CR/PR vs. SD 6
weeks p=0.029; 3 mths p=0.019) than tamoxifen cases with similar degree of
ER fall. These findings suggest that GLA may enhance the effects of tamoxifen-
induced ER down-regulation to produce a superior clinical response.

Reouse6wks p=0_0I Chin2  Rcspomnse 3 ms  p0.016 Chi2

Thrp      PR(%w) I SD%    P D(Y.) CR()    |  %     D(/)P %
Tun       4 (5)   40 (5)   3 (6.4)  0      6 (13)  3S (8(1)  3 (6.4)
T+G1A     12 (31)  26(63)  0       2 (53)  14 (37) 21(55)  1(2.6)

(CR-vonq~eic  PR=p tz i rmpoas,  SD=Uatic disase  PD=Pwogressivc disae

Table: GIkcl respone by treatm at 6 weeks ad 3 saths

Comelusions. Our results propose GLA as a useful adjunct to primary tamoxifen
in endocrine sensitive breast cancer. Continued follow-up will determine
whether this faster initial response will translate into longer ultimate duration of
control.

p66           3D DOSE DISTRIBU      ONS IN TANGENTIAL FIELD

IRRADIATION   OF THE     INTACT   BREAST   USING
WEDGES AND INDIDUAL COMPENSATORS.

U. Brown, AT. Redpath & .H. Kunkler, Cinical Oncogy, Westem General
Hospital, Edinburgh, Scotland.

Variation in dose thmogeneity (DH) in tangenal breast irradiation may be up
to 25%. DH may be important to local conto and cosmesis. The purpose of
this study is to assess (i) the DH in 3D in the breast iradiated with wedged

angential fields, and (ii) the     t achievable using compensators.

17 patients were CT scanned using contiguous 1cm slices from =2cm
supenor and inferior to the field edges. The dose distitions were calculated
using a 3D akgonthm that calculates pnrmary and scatter dose separately
using a differential scatter air ratio method, and corrects scatter dose for the
presence of h

The DH is assesd using diffeental dose volume histogramsn and
quantified by the diffence between the highest isodose which just covers
the iradiated breast volume, and the maxdmumn sodose with at least one
dion      >1.5cm. The resultng % variation in dose within the breast is
shown in Table 1.

The overal vanation is split into the transverse component (Trans.), which is
the imting vaation, and the longitudinal component (Long.) in which the
greatest improvement is seen.

Table 1

WEDGES

COMPENSATORS

Trans.   Long.   Overall        Trans.   Long.   Overall
Av.     72       5.9     13.1           60       2.2      82
S.D.    26       24       2.6           25       14      2.2
Max      13      12       19            13       6        14
Min      3        2       10             3       1        5

Concs      - Dose hornogenety could be reduced to as little as 5% using
xiiidual conpensators, with potetal benefits in local control and cosrresis.

46 Poster Presentations

RADIATION INDUCED BRACHIAL PLEXOPATHY
P69            AFTER AXAL^    AT       FOR EAIY

BREAST CANCER I.H-Krkeler1), N. Mder (1), G Kerr
(1), R. Grant (2). Dept do Clinical Oncobgy (1) and Clinical Neurosciences (2),
Westem General Hospital, Crewe Rd, Edinbrgh, EH4 2XU.

In Edinburgh from 1980 until 7/90 patients receiving perpheral

lymphatic irradiation for early breast cancer were treated supine
with the ipsilateral arm abducted by (a) computer planned

tangential fields to the breast/chest wall and (b) an anterior

shoulder field covering the axilla and supraclavicular fossa with
a posteror axillary boost(PAB). All fields were treated daily (45

Gy to mid axilla in 20 fr. over 4 weeks[4-6MV photons]) The daily
dose from the posterior axillary boost was approx 45cGy. No
case of radiation induced brachial plexopathy (RIBP )was

reported among patients treated with axillary irradiation and a
daily PAB. From 8/90 the posterior axilla field was treated on
altemate days (daily dose approx 90 cGy). A retrospective

survey was undertaken of the case notes of 223 consecutive
patients treated between 8/90-7/91 by postoperative

radiotherapy to the peripheral lymphatics following wide excision
or mastectomy for TO-3,NO-1 IMO breast cancer. The case

records of patients with clinical features suspicious of RIBP were
reviewed by a neurologist (RG). Maximum axillary dose and the
volume of the shoulder field were recorded. Median follow up
was 67.8 months.25% of the cohort died of breast cancer. 9
remain alive post relapse. Two disease free patients mnet the

criteria of RIBP (0.9%). CT(Case 1) and MR1 of the axilla (Case
2) were negative. In each case of RIBP (i) the maximum axillary
dose was less than the mean maximum axillary dose (5224.9
cGy) for the whole cohort. (ii) the volume of the shoulder field
irradiated was less than the mean of 11 92.8cm.Conclusion:

there has been a slight but non statistically significant rse in the
incidence of RIBP with a change from daily to altemate day

posterior axillary boost. It does not appear to relate to higher
axillary dose or volume of axillary irradiation.